# Ramifications of *POU4F3* variants associated with autosomal dominant hearing loss in various molecular aspects

**DOI:** 10.1038/s41598-023-38272-w

**Published:** 2023-08-03

**Authors:** Sang-Yeon Lee, Min Young Kim, Jin Hee Han, Sang Soo Park, Yejin Yun, Seung-Cheol Jee, Jae Joon Han, Jun Ho Lee, Heeyoung Seok, Byung Yoon Choi

**Affiliations:** 1 Department of Genomic Medicine, Seoul National University Hospital, Seoul National University College of Medicine, Seoul, Republic of Korea; 2 Department of Otorhinolaryngology-Head and Neck Surgery, Seoul National University Hospital, Seoul National University College of Medicine, Seoul, Republic of Korea; 3 Department of Otorhinolaryngology-Head and Neck Surgery, Seoul National University Bundang Hospital, Seoul National University College of Medicine, 300 Gumi-dong, Bundang-gu, Seongnam, 463-707 Republic of Korea; 4 Department of Transdisciplinary Research and Collaboration, Genomics Core Facility, Biomedical Research Institute, Seoul National University Hospital, Seoul, Republic of Korea; 5 Sensory Organ Research Institute, Seoul National University Medical Research Center, Seoul, Republic of Korea

**Keywords:** Genetics, Medical research, Molecular medicine, Signs and symptoms

## Abstract

*POU4F3*, a member of the POU family of transcription factors, commonly causes autosomal dominant deafness. Exome sequencing was used to identify four novel variants in *POU4F3* (NM_002700.2), including c.564dupA: p.Ala189SerfsTer26, c.743T > C:p.Leu248Pro, c.879C > A:p.Phe293Leu, and c.952G > A:p.Val318Met, and diverse aspects of the molecular consequences of their protein expression, stability, subcellular localization, and transcriptional activity were investigated. The expression of three mutant proteins, encoded by missense variants, was reduced compared to the wild-type protein, demonstrating that the mutants were unstable and vulnerable to degradation. Additionally, all the mutant proteins had distinct subcellular localization patterns. A mutant protein carrying p.Ala189SerfsTer26, in which both mono- and bi-partite nuclear localization signals were disrupted, showed abnormal subcellular localization. Resultantly, all the mutant proteins significantly reduced the transcriptional activity required to regulate the downstream target gene expression. Furthermore, we identified the altered expression of 14 downstream target genes associated with inner ear development using patient-derived lymphoblastoid cell lines. There was a significant correlation of the expression profile between patient-derived cells and the cochlear hair cells, which provided a breakthrough for cases where the collection of human cochlear samples for transcriptome studies was unfeasible. This study expanded the genotypic spectrum of *POU4F3* in DFNA15, and further refined the molecular mechanisms underlying *POU4F3*-associated DFNA15.

## Introduction

*POU4F3* (NM_002700; NP_002691) comprises two exons located on chromosome 5q32^1^, encoding a member of the POU family of transcription factors with two highly conserved POU domains: a POU-specific domain and a POU homeodomain^2^. These domains bind a target DNA octamer that displays a conserved consensus sequence^2^. The function of *POU4F3* as a transcriptional regulator depends on the intact function of both POU domains^2,3^. Specifically, *POU4F3* protein is highly expressed in cochlear and vestibular hair cells in the inner ear, where it is involved in the maturation, differentiation, and survival of these cells^4,5^. It was recently reported that *POU4F3* heterozygous knock-in (Pou4f3^Δ/+^) and knock-out (Pou4f3^−/+^) mice developed progressive hearing loss concomitant with the apparent loss of outer hair cells, mirroring the human dominant form of deafness (DFNA15)^6^. In humans, *POU4F3* is one of the most common autosomal-dominant deafness-associated genes, variants of which cause post-lingual onset, progressive non-syndromic hearing loss (NSHL)^7–13^. Previous studies suggested that *POU4F3* is one of the signature gene associated with mid-frequency hearing loss, showing a down-sloping configuration over time^10,12^. More than 30 *POU4F3* heterozygous variants, mostly in their DNA-binding POU domains, have been reported to cause DFNA15 in the literature, with the phenotypic variability in terms of age of onset, severity, and audiogram configuration^7–13^. Nonetheless, evidence on genotype–phenotype correlation and molecular mechanisms remains limited, despite the clinical and scientific importance of *POU4F3* hair-cell specific transcriptional factor in the context of NSHL.

In this study, we identified four novel *POU4F3* variants segregating as a dominant trait in four unrelated Korean families. We characterize all four novel *POU4F3* variants functionally via computational structural modeling and functional studies, revealing diverse pathogenic mechanisms underlying *POU4F3*-associated hearing loss. Furthermore, we investigate whether *POU4F3* variants can impact the expression of downstream target genes, potentially affecting inner ear development, using patient-derived cell lines.

## Results

### Identification and classification of novel *POU4F3* variants

**Figure 1:**
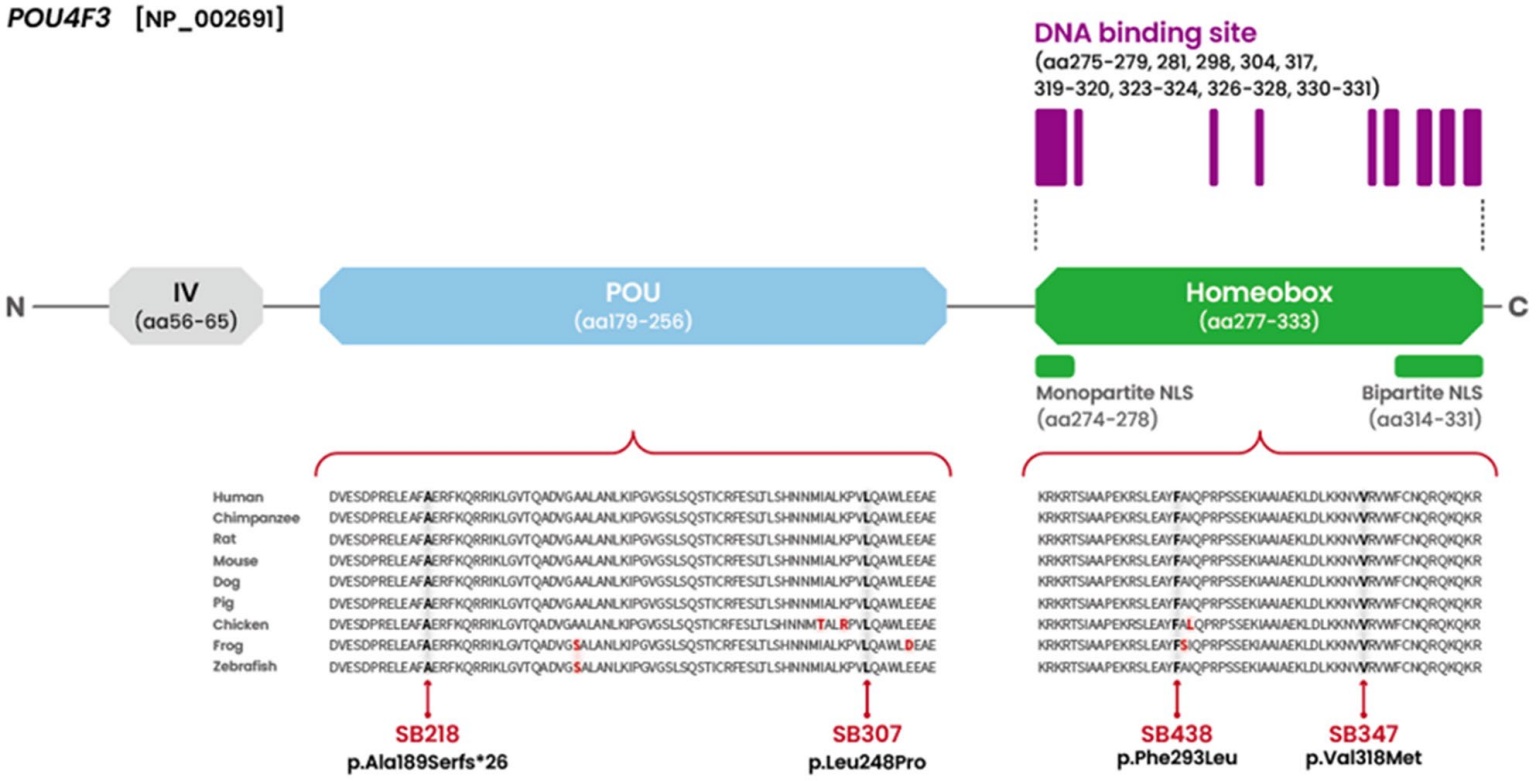
Four novel *POU4F3* variants within the functional DNA-binding domains. Two (p.Ala189SerfsTer26 in SB218 and p.Leu248Pro in SB307) were located in the POU-specific domain, while the remaining two (p.Phe293Leu in SB438 and p.Val318Met in SB347) were in the POU-homeodomain. Conservation of the affected residues among species was documented for all *POU4F3* variants identified in the study.

Exome sequencing revealed four novel *POU4F3* variants (NM_002700.2) not previously reported in the literature, segregating as a dominant trait in the four unrelated Korean families: one frameshift variant produced a premature termination codon in the POU-specific domain (c.564dupA:p.Ala189SerfsTer26) that lacked both mono- and bi-partite nuclear localization signals (NLSs); two missense variants (c.743T > C:p.Leu248Pro and c.879C > A:p.Phe293Leu) in which the alterations were located within the POU-specific domain and POU homeodomain, respectively, but outside the NLSs; and one missense variant (c.952G > A:p.Val318Met) in which the alteration was located within the bipartite NLS (Fig. 1). Co-segregation of the variants with the phenotypes of the family members, including both parents, was confirmed. The novel frameshift variant (p.Ala189SerfsTer26) produced a truncated protein, which was predicted to escape the nonsense-mediated mRNA decay (NMD). The variant was not present in any population databases. The Ala189 residue produced by *POU4F3* orthologs across various species is well preserved (http://genome.ucsc.edu/) (Fig. 1). Furthermore, in silico analyses, including combined annotation dependent depletion (CADD) phred (https://cadd.gs.washington.edu/) and rare exome variant ensemble learner (REVEL) (https://sites.google.com/site/revelgenomics/) scores, consistently indicated that this variant is disease-causing. Next, a novel missense variant (p.Leu248Pro), located in the C-terminus of the POU-specific domain, was extremely rare, meeting the PM2 criteria as per the American College of Medical Genetics and Genomics/Association for Molecular Pathology (ACMG/AMP) guidelines for hearing loss. Moreover, the remaining two novel missense variants (p.Phe293Leu and p.Val318Met), located in the POU-specific domain and POU homeodomain, respectively, were absent from population databases. These three missense variants were consistently predicted to be disease-causing by in silico analyses, as evidenced by CADD phred and REVEL scores, and the corresponding residues in proteins encoded by *POU4F3* orthologs between several species are highly conserved. Accordingly, based on ACMG/AMP guidelines for hearing loss, our novel *POU4F3* variants were classified as “pathogenic” (p.Ala189SerfsTer26) and “variant of uncertain significance” (p.Leu248Pro, p.Phe293Leu, and p.Val318Met) (Table 1).

**Table 1:** Novel *POU4F3* variants in the current study and in-silico prediction analysis

Proband	Genomic position: change (GRCh37/hg19)	HGVS		Location (exon/domain)	Zygosity/inheritance	Insilico predictions		Alternative allele frequency		ACMG/AMP 2018 guideline	
		Nucleotide change	Amino acid change			CADD Phred	REVEL	KRGDB (1722 individuals)	GMAF (gnomAD)	Criteria	Classification
SB218-423	Chr5:145719554A>AA	c.564dupA	p.Ala189SerfsTer26	Exon2/POU	Het/autosomal dominant	NA	NA	Absent	Absent	PVS1, PM2, PS3_supporting	Pathogenic
SB307-610	Chr5:145719733T>C	c.743T>C	p.Leu248Pro	Exon2/POU	Het/autosomal dominant	29.3	0.950	Absent	Absent	PM2, PP3, PS3_supporting	VUS
SB438-852	Chr5:145719869C>G	c.879C>A	p.Phe293Leu	Exon2/homeobox	Het/autosomal dominant	24.8	0.913	Absent	Absent	PM2, PP3, PS3_supporting	VUS
SB347-679	Chr5:145719942G>A	c.952G>A	p.Val318Met	Exon2/homeobox	Het/autosomal dominant	29.6	0.936	Absent	Absent	PM2, PP3, PS3_supporting	VUS

Refseq transcript accession number NM_002700.2; Refseq protein accession number NP_002691.

HGVS: Human Genome Variation Society (https://www.hgvs.org/).

Sequence Variant Nomenclature (https://mutalyzer.nl/).

CADD: combined annotation dependent depletion (https://cadd.gs.washington.edu/).

REVEL: rare exome variant ensemble learner (https://sites.google.com/site/revelgenomics/).

KRGDB: Korean reference genome database (http://152.99.75.168:9090/KRGDB/welcome.jsp).

gnomAD: the genome aggregation database (https://gnomad.broadinstitute.org/).

*MAF* minor allele frequency, *Het* heterozygote, *VUS* variant uncertain significance, *NA* not available.

### Clinical phenotypes

**Figure 2:**
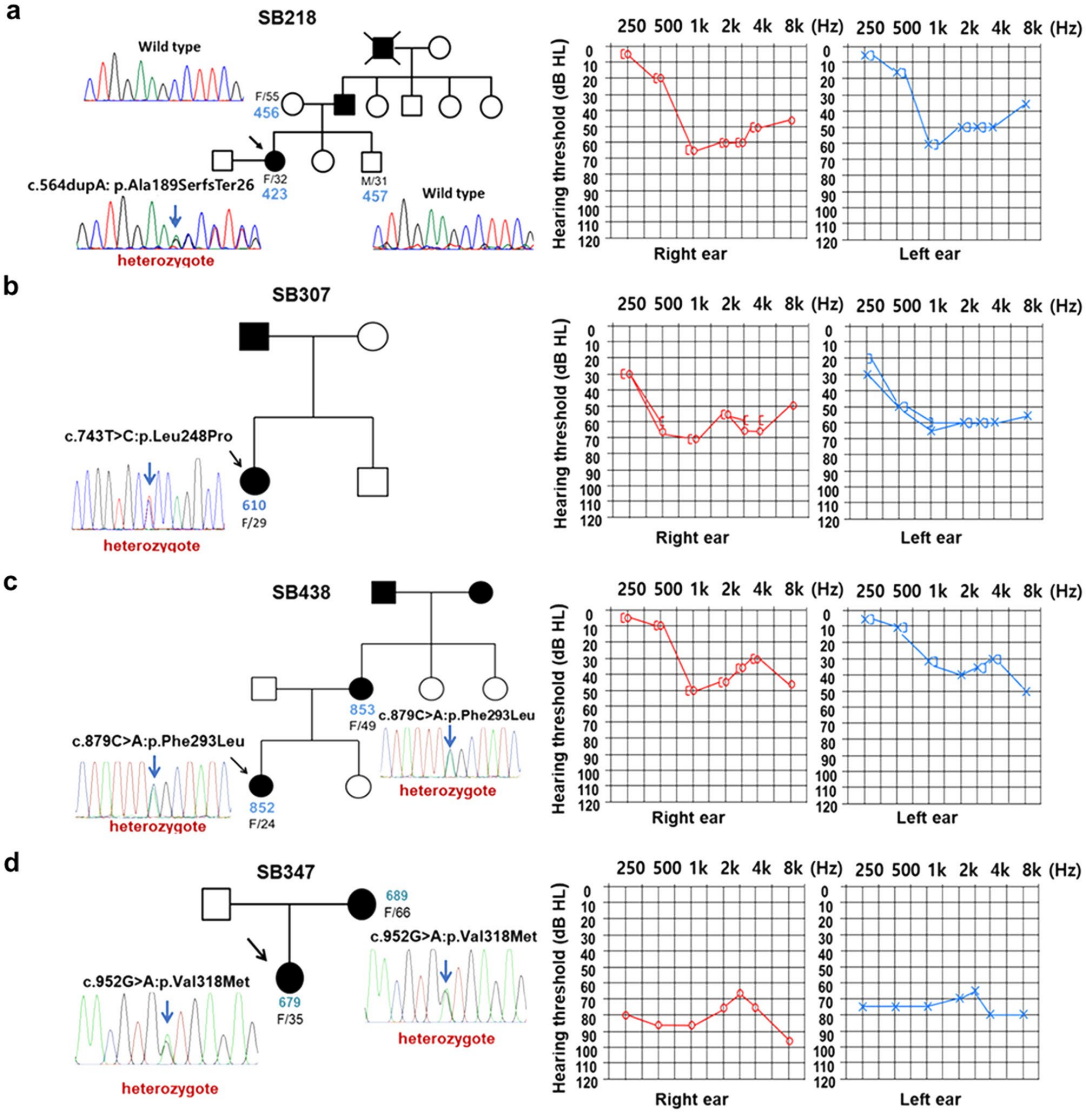
Pedigrees of the four families, Sanger sequencing traces of the respective *POU4F3* variants, and the audiological phenotypes of the affected individuals. The pedigrees of the Sanger sequence chromatograms of the four families, exhibiting segregation of p.Ala189SerfsTer26 in SB218 (**a**), p.Leu248Pro in SB307 (**b**), p.Phe293Leu in SB438 (**c**), and p.Val318Met in SB347 (**d**). The audiograms of SB218, SB307, and SB438 were associated with moderate NSHL with mid-specific hearing loss configuration, while that of SB347 indicated severe-to-profound NSHL with flat configuration.

Diverse audiological phenotypes, in terms of degree of severity and audiogram configuration, were observed (Fig. 2). In family SB218, proband SB218–423 (32 years old at ascertainment) initially demonstrated symmetrical moderate to severe NSHL, including predominant mid-frequency hearing loss (symmetric notch at 1 kHz up to 60 dB HL in both ears), with a down-sloping configuration (Fig. 2a). Affected individuals in this family, including the proband’s father and grandfather, had experienced progressive NSHL since their late 20 s. Specifically, the proband's father planned to undergo cochlear implantation due to progressive deterioration of hearing. In family SB307, proband SB307–610 (29 years old at ascertainment) initially demonstrated symmetrical moderate-to-severe NSHL, including a predominant mid- and high-frequency hearing loss, with a down-sloping configuration in the left ear (Fig. 2b). The affected individual (SB307–610) received a middle ear implant, but the hearing loss gradually progressed. Hearing status of the proband’s father was allegedly poor but had not been documented. In family SB438, proband SB438–852 (24 years old at ascertainment) initially demonstrated mild to moderate NSHL, including predominant mid-frequency hearing loss in the right ear (at 1 kHz up to 50 dB HL), with a down-sloping configuration in the left ear (Fig. 2c). She was using hearing aids because of gradually progressive hearing deterioration at middle and higher frequencies. In family SB347, proband SB347–679 (35 years old at ascertainment) unexpectedly demonstrated severe to profound NSHL (more than 70 dB HL across all frequencies), with a flat configuration in the both ears (Fig. 2d). The age of onset was the late teens. Over a 10-year follow-up period, SB347–679 experienced rapid progressive deterioration of bilateral hearing across all frequencies, as well as a decrease in speech discrimination scores. She underwent bilateral cochlear implantation using a slim modiolar electrode (CI632; Cochlear, Australia) via a round-window approach. Compared to the preoperative speech evaluation, speech perception scores improved significantly by 3 and 6 months postoperatively, and the effects were longitudinally sustained. The proband's mother had also experienced progressive bilateral hearing loss since the age of 20 years, and had undergone cochlear implantation 15 years previously. Medical histories and general physical examinations of all affected individuals were otherwise unremarkable. Specifically, none of them complained of vertigo or dizziness. The radiological evaluation, including temporal bone computed tomography and internal acoustic canal magnetic resonance imaging, did not reveal any inner ear pathology or brain lesion that might have precipitated hearing loss in the probands.

### 3D protein structure

**Figure 3:**
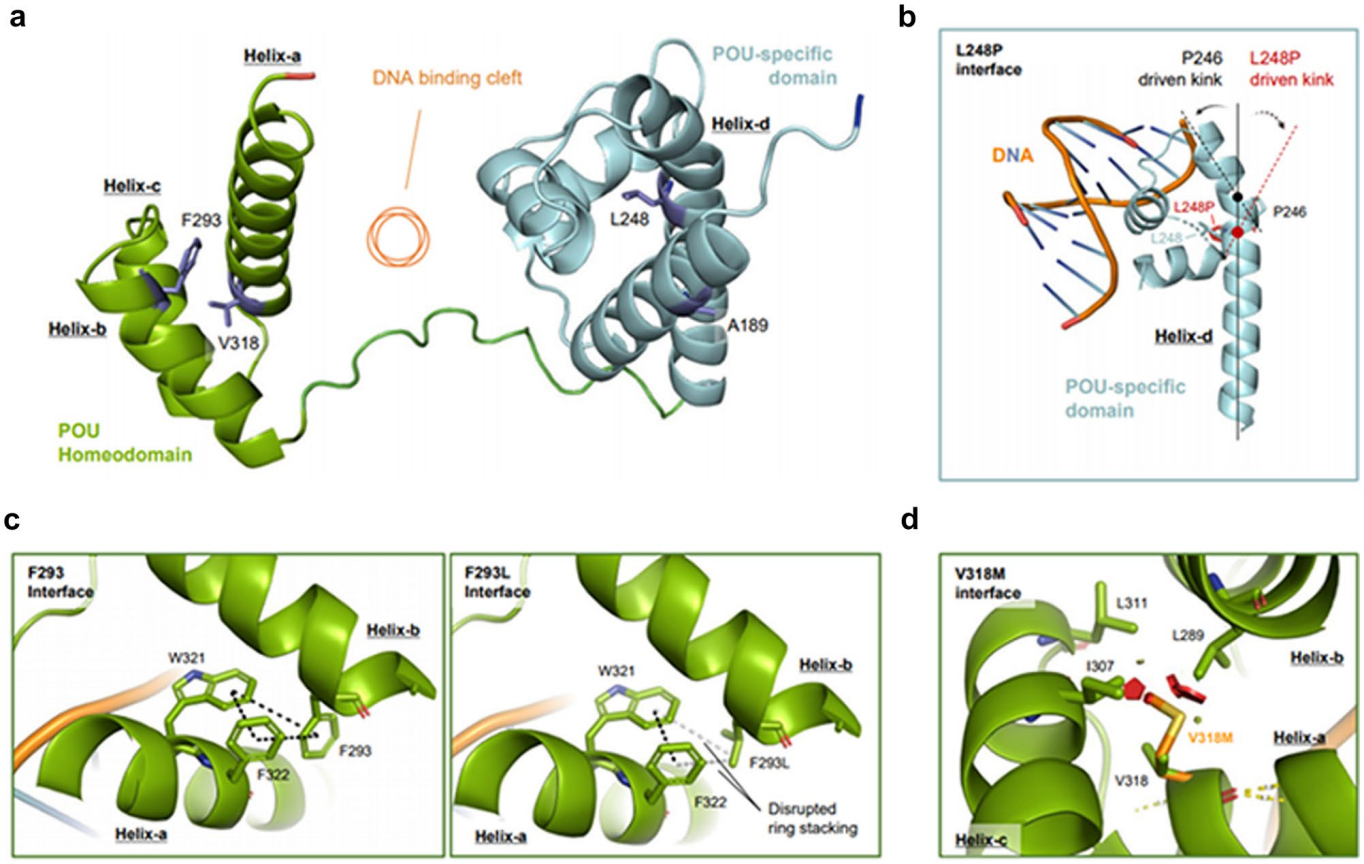
Novel *POU4F3* variants destabilize the inter-helical interactions, impairing the transcriptional activity of *POU4F3*. (**a**) Sideview of Alphafold generated model structure of *POU4F3*. (Jumper et al.^37^ Highly accurate protein structure prediction with AlphaFold., Nature). POU homeodomain (green) and POU-specific domain (cyan) assembled with DNA binding cleft (orange circle) in between. Val318 and Phe293 are present in the Helix-a and Helix-b of the homeodomain (green), respectively, while Leu248 is in the Helix-d of the POU-specific domain (cyan). All the mutant residues are facing intra-helical spaces, not directly interacting with DNA. (**b**) Intra-helical proline substitution at Leu248 causes helical kinks. A 27-amino acid long helix-d has a natural kink (black dotted line) driven by Pro246 in the middle. Additional proline substitution induces the formation of an additional kink (red dotted line) starting from p.Leu248Pro (red dot), causing dramatic conformational changes in the POU-specific domain. (**c**) Phe293 forms aromatic ring stacking (black dotted line) with Trp321 and Phe322 in helix-a (left), stabilizing the interhelical interface. The p.Phe293Leu variant largely disrupts biochemical interactions between helix-a and helix-b, destabilizing helical assembly of POU-homeodomain. (**d**) Key amino acid residues of intramolecular hydrophobic cavity of POU homeodomain. Val318 forms hydrophobic interactions with Ile307, Leu289, and Leu311. The Val318Met mutant with long side chain clashes (red polygons), with the adjacent Ile307 and Leu289, changing the distance between helices. *L* Leu, *V* Val, *P* Pro, *F* Phe, *W* Trp, *I* Ile, *M* Met.

The DNA binding interface of Alpha-fold generated *POU4F3* model structure is depicted in Fig. S1. Alpha-fold generated model structure of *POU4F3* was used to examine the effects of the identified variants on *POU4F3* protein structure, compared to wild-type *POU4F3* protein (Fig. 3a). Leu248 amino acid residue is present in the helix-d of the POU-specific domain. Intra-helical proline substitution at Leu248 causes helical kinks, resulting in dramatic conformational changes in the POU-specific domain (Fig. 3b). Next, Phe293 amino acid residue is present in the helix-b of the POU-homeodomain. The missense variant p.Phe293Leu collapses the interhelical interface (aromatic ring stacking) between Phe293, Trp321, and Phe322 in helix-a (left) by disrupting biochemical interactions between helix-a and helix-b, which in turn destabilize the helical assembly of POU-homeodomain (Fig. 3c). Val318 amino acid residue is present in the helix-a of the POU-homeodomain. The long side chain of the missense variant p.Val318Met collapses the hydrophobic interactions with Ile307, Leu289, and Leu311, and induces hydrogen-bonding, destabilizing the POU-homeodomain helical assembly by causing molecular clashes with the adjacent Ile307 and Leu289 (Fig. 3d). Predicted aligned error (PAE) score, which reflects inter-domain accuracy, also indicated that p.Ala189SerfsTer26 variant-induced premature termination of translation destroys the *POU4F3* protein structure, including the DNA-binding functional domains, in turn destabilizing the protein (Fig. S2). Collectively, the *POU4F3* variants compromised protein stability, and probably impaired the DNA-binding ability.

### Protein expression and stability

**Figure 4:**
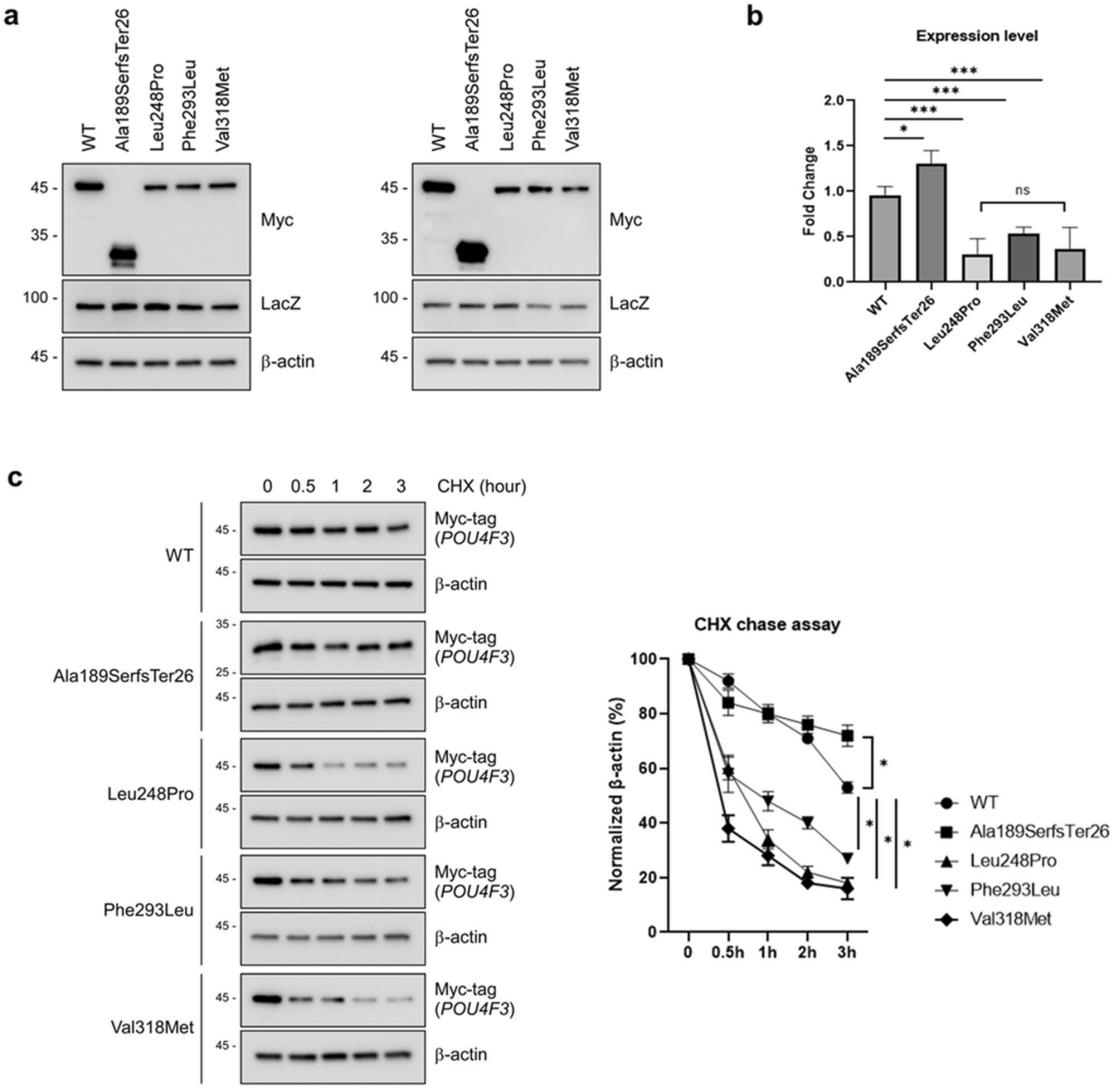
Western blot analysis for *POU4F3* wild-type, frameshift, missense variants by transient transfection at HEK293T cell. (**a**) Expressions of *POU4F3* wild-type and mutants were detected by western blotting in HEK 293T cells. Molecular weight of wild-type and mutant proteins (p.Leu248Pro, p.Phe293Leu, and p.Val318Met) are 36 kDa, whereas molecular weight of truncated mutant protein (p.Ala189SerfsTer26) is 21 kDa. The immunoblots are representative of independent repetitive experiments. LacZ is used as a transfection control. (**b**) The bands intensity was quantified by Image J. The band intensity was normalized to β-actin. Intensity data was presented as means ± standard deviations from two independent plots in a triplicate manner. Consequently, the sample size for each experiment was six. (**c**) Comparison of the stability of wild-type and mutant *POU4F3* using protein stability assays in the transient overexpression system. HEK293 cells, overexpressing *POU4F3*, were treated with cycloheximide (80 µg/ml) for up to 3 h to block the general translation. The CHX chase assay experiment was performed once, with three measurements taken during the process. As a result, the sample size for each experiment was three. WT, wild type; LacZ, transfection control; β-actin, loading control; ns, no statistical significance; *p < 0.05, ***p < 0.001, one-way ANOVA with Bonferroni comparisons. The original immunoblots (uncropped, full length membranes with membrane edges visible, and standard protein size markers and expected molecular weight labeled) matched to the cropped versions (this figure) were all provided in Fig. S3.

The western blot analysis demonstrated that the wild-type and the three mutant proteins carrying missense variants (p.Leu248Pro, p.Phe293Leu, and p.Val318Met) were expressed as a single band corresponding to the correct molecular weight (36 kDa), indicating that the staining was derived explicitly from the tagged *POU4F3* proteins (Fig. 4a). Compared to the wild-type protein, the three mutant proteins carrying missense variants had weaker intensities, probably due to protein instability (Fig. 4b). Additionally, the p.Ala189SerfsTer26 *POU4F3* protein was stably expressed with a smaller molecular weight (21 kDa) due to premature termination of the *POU4F3* protein. Interestingly, the expression of a truncated protein (p.Ala189serfs*26) was stronger than the wild-type protein (Fig. 4a,b). To determine whether *POU4F3* variants destabilize *POU4F3* protein, we performed cycloheximide (CHX) chase assays to block protein synthesis. HEK293T cells were transfected with wild type and four mutant *POU4F3* vectors for 24 h, followed by treatment with CHX (80 µg/ml) for 1, 2, and 3 h, respectively. Three missense variants decreased the stability of *POU4F3* protein compared with wild-type protein. Conversely, the half-life of the truncated mutant protein (p.Ala189SerfsTer26) lacking both mono- and bi-partite NLSs showed a longer trend compared with the wild-type protein, suggesting the mutant protein (p.Ala189SerfsTer26) was more stable than the wild-type protein (Fig. 4c). The original immunoblots (uncropped, full-length membranes with membrane edges visible) were provided in Fig. S3.

### Transcriptional activity

**Figure 5:**
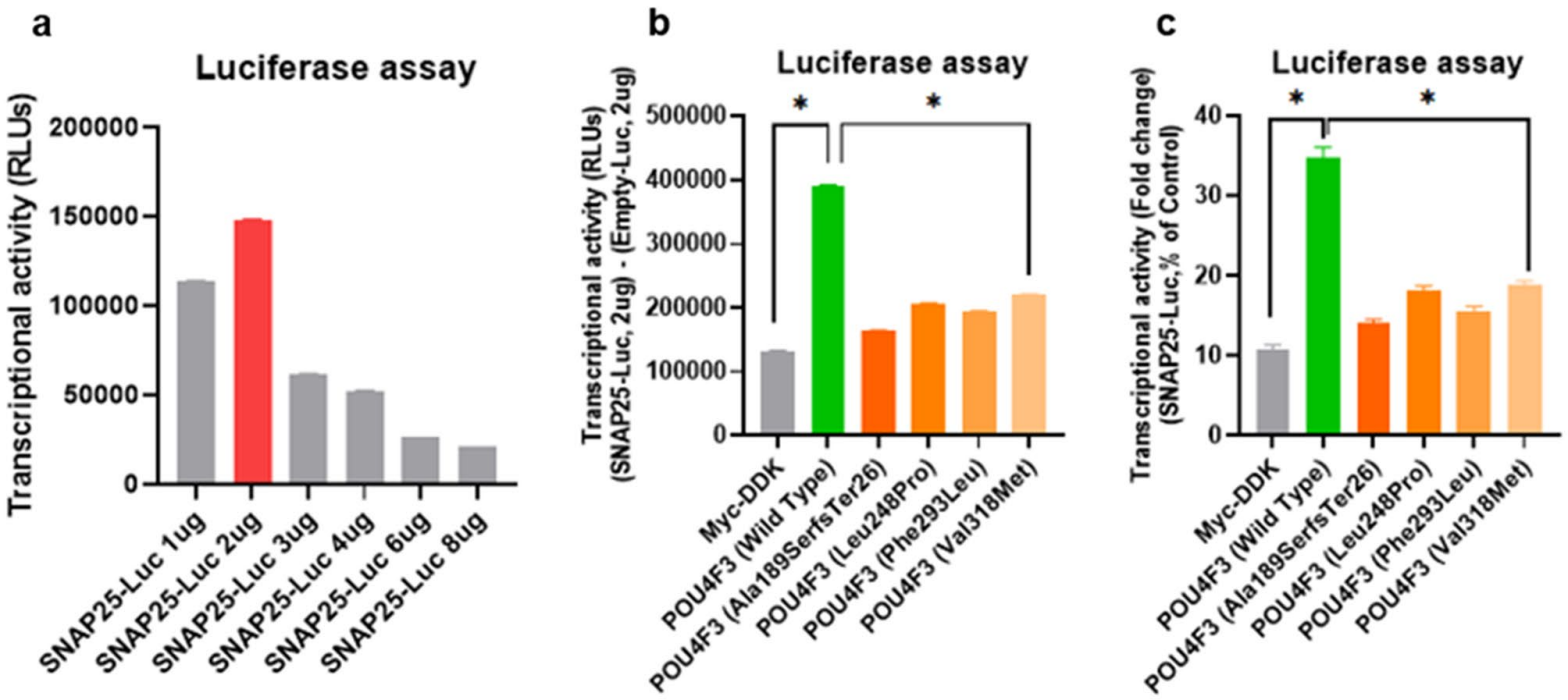
Transcriptional activity of novel *POU4F3* variants. (**a**) Luciferase activities measured under specific conditions. To minimize the ceiling effect, the condition (i.e., empty‐Luc 2 µg and SNAP25‐Luc 2 µg) was determined as the luciferase vector system. (**b**,**c**) The transcriptional activities of the wild-type and mutant *POU4F3* proteins in the SNAP25-Luc vector were normalized to that of the internal control (Myc-DDK). Using the luciferase vector system, the transcriptional activity in the wild-type and the four *POU4F3* variants were analyzed. All variants exhibited significantly reduced transcriptional activities compared to the wild type. Experiments were conducted in duplicate, and measurements were taken three times to determine luciferase activity. Consequently, the sample size for each experiment was six. The four mutant constructs corresponding to each variant differentiated by employing distinct colors for clear visualization. *, a statistical significance by one-way ANOVA and Bonferroni’s multiple comparison test.

To investigate the transcriptional activities of the mutant *POU4F3* proteins, an in vitro luciferase reporter assay incorporating the *SNAP-25*-Luc reporter construct was performed (Fig. S4). HEK293T cells were transfected with six pCMV6 plasmid constructs encoding Myc-DDK only (negative control), wild-type *POU4F3*, mutant *POU4F3* (p.Ala189SerfsTer26), mutant *POU4F3* (p.Leu248Pro), mutant *POU4F3* (p.Phe293Leu), and mutant *POU4F3* (p.Val318Met) (Fig. 5). We then compared the fold changes in the luciferase activities of mutant *POU4F3* proteins to those of the wild-type protein, normalized to those of the internal control (Myc-DDK). The experimental condition yielding optimal wild-type *POU4F3*-induced transcription efficiency was determined to be 2 μg *SNAP-25-Luc*, to minimize the influence of the ceiling effect (Fig. 5a). While the wild-type *POU4F3* increased the luciferase activity approximately four-fold changes, the mutant *POU4F3* showed only two-fold increase in luciferase activity, demonstrating a significantly poorer transcriptional activity of mutant *POU4F3* (*p* < 0.001) to elicit transcription of the downstream target genes of *POU4F3* (Fig. 5b,c).

### Subcellular localization

**Figure 6:**
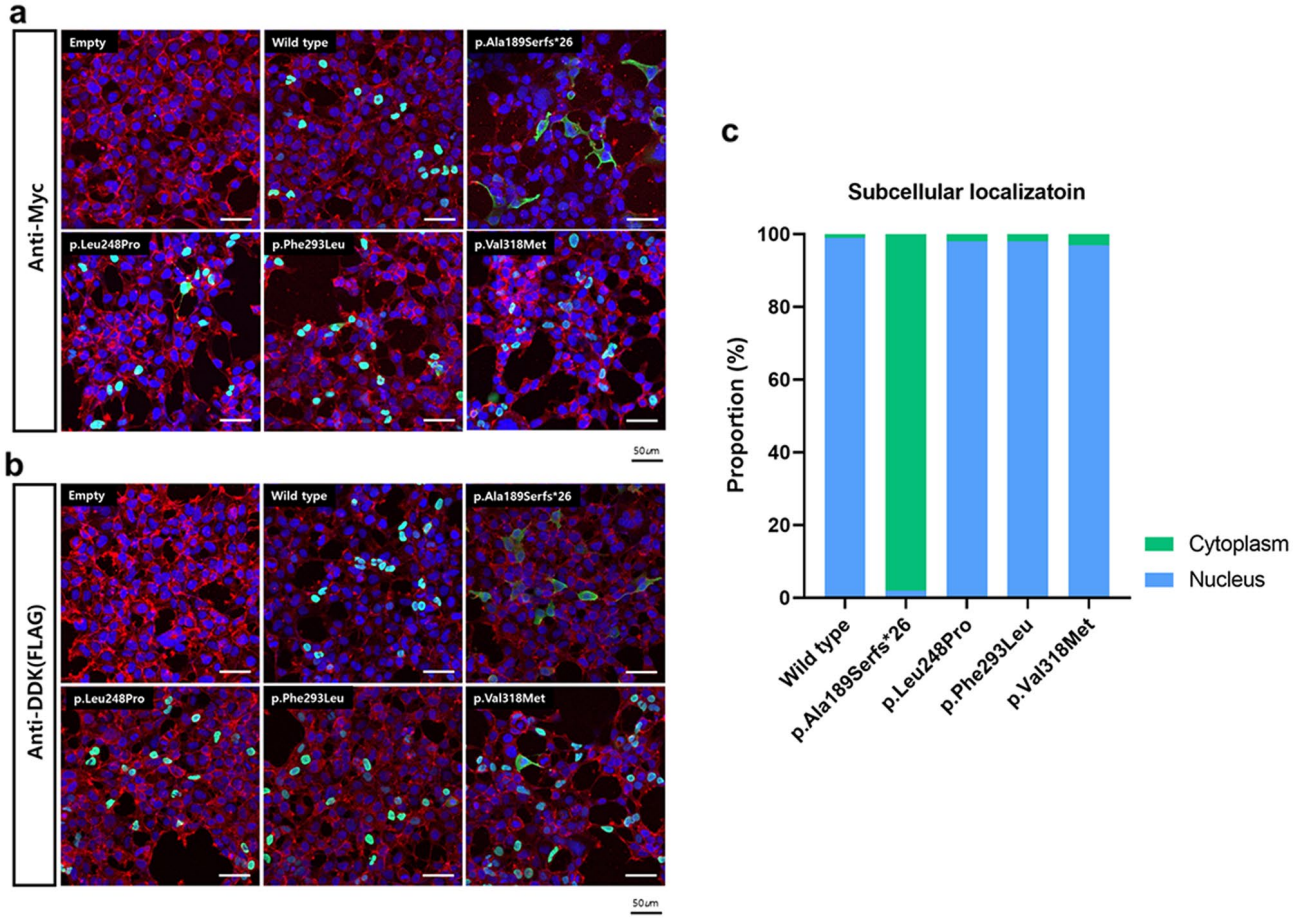
Immunofluorescence of the wild-type and mutant *POU4F3* proteins. (**a**) Cells were immuno-stained with anti-Myc (green) and phalloidin (red). The nuclei were stained with DAPI (blue). (**b**) Cells were immuno-stained with anti-DDK (green) and Rhodamine-phalloidin (red). Rhodamine-phalloidin (red) staining was used to label F-actin and stabilize actin filaments in vitro. The nuclei were stained with DAPI (blue). Upon examination through confocal microscopy, the region where the green fluorescence (representing the target protein) and the blue fluorescence (DAPI-stained nuclei) overlap appears as a turquoise color. (**c**) Quantitation of cytoplasmic and nuclear localization of *POU4F3*, depending on the variants.

Subcellular localization of transcriptional factors in the nucleus is necessary for its transcriptional activity that regulates target gene expression. All the mutant *POU4F3* proteins showed significantly reduced reporter gene expression compared to the wild-type protein. The HEK293T cells transfected with the empty vector (negative control) demonstrated no cytoplasmic or nuclear fluorescence, confirming that the small tags attached to empty vectors did not induce any additional trafficking of the cloned protein to the target cells and organelles (Fig. 6a,b). Further, HEK293T cells were transfected with constructs encoding wild-type and mutant proteins fused to C-terminal Myc-DDK tags. Notably, the mutant *POU4F3* (p.Ala189SerfsTer26), which lacks both mono- and bi-partite NLSs, localized to both the cytoplasm and the nucleus, but the nuclear proportion (approximately 2%) was significantly lower than that of the cytoplasm (approximately 98%) (Fig. 6a,b). In contrast, all other mutant *POU4F3* proteins (p.Leu248Pro, p.Phe293Leu, and p.Val318Met) localized exclusively to the nuclei (approximately 98%), consistent with the localization of the wild-type protein (Fig. 6c).

### RNA sequencing and bioinformatic analyses

**Figure 7:**
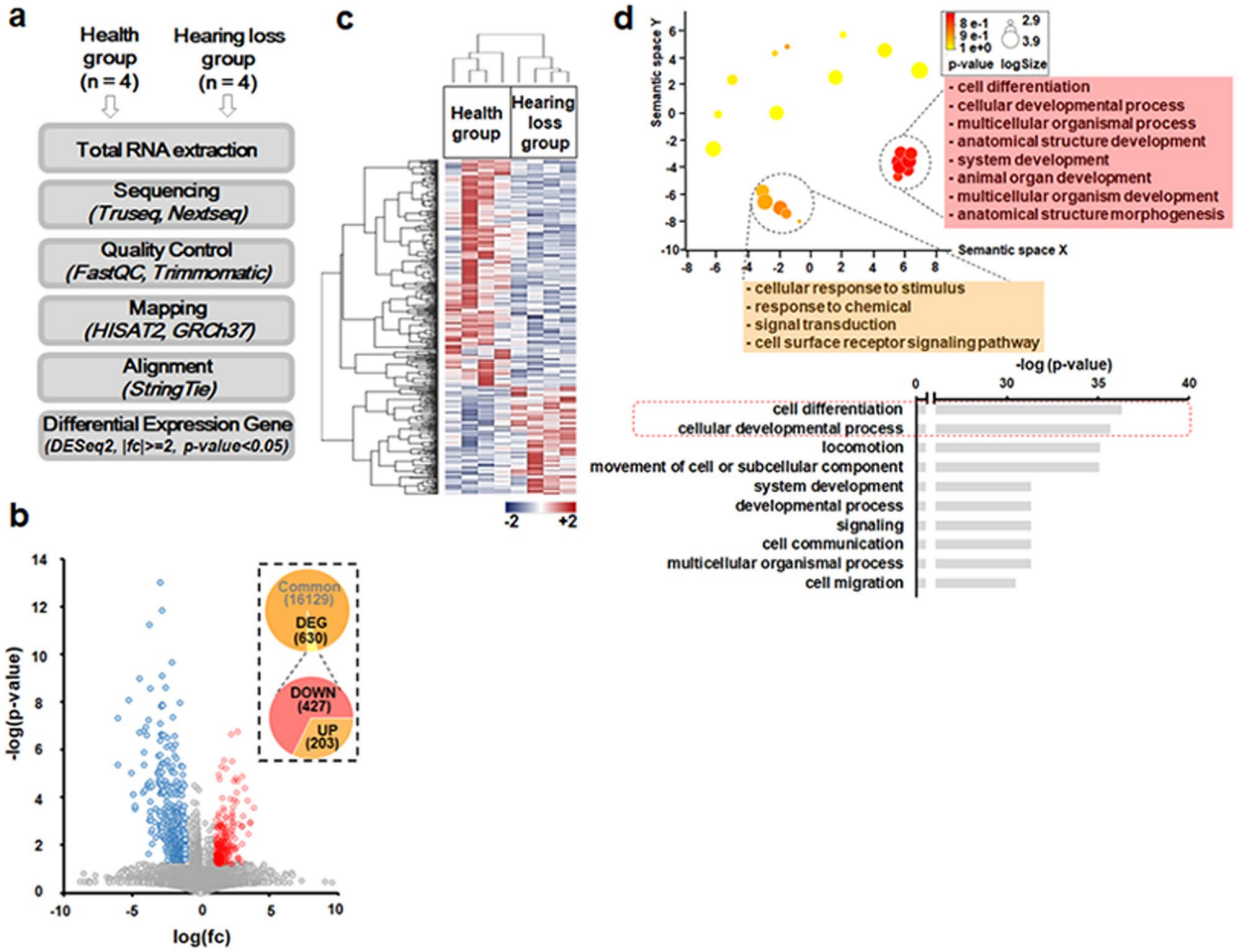

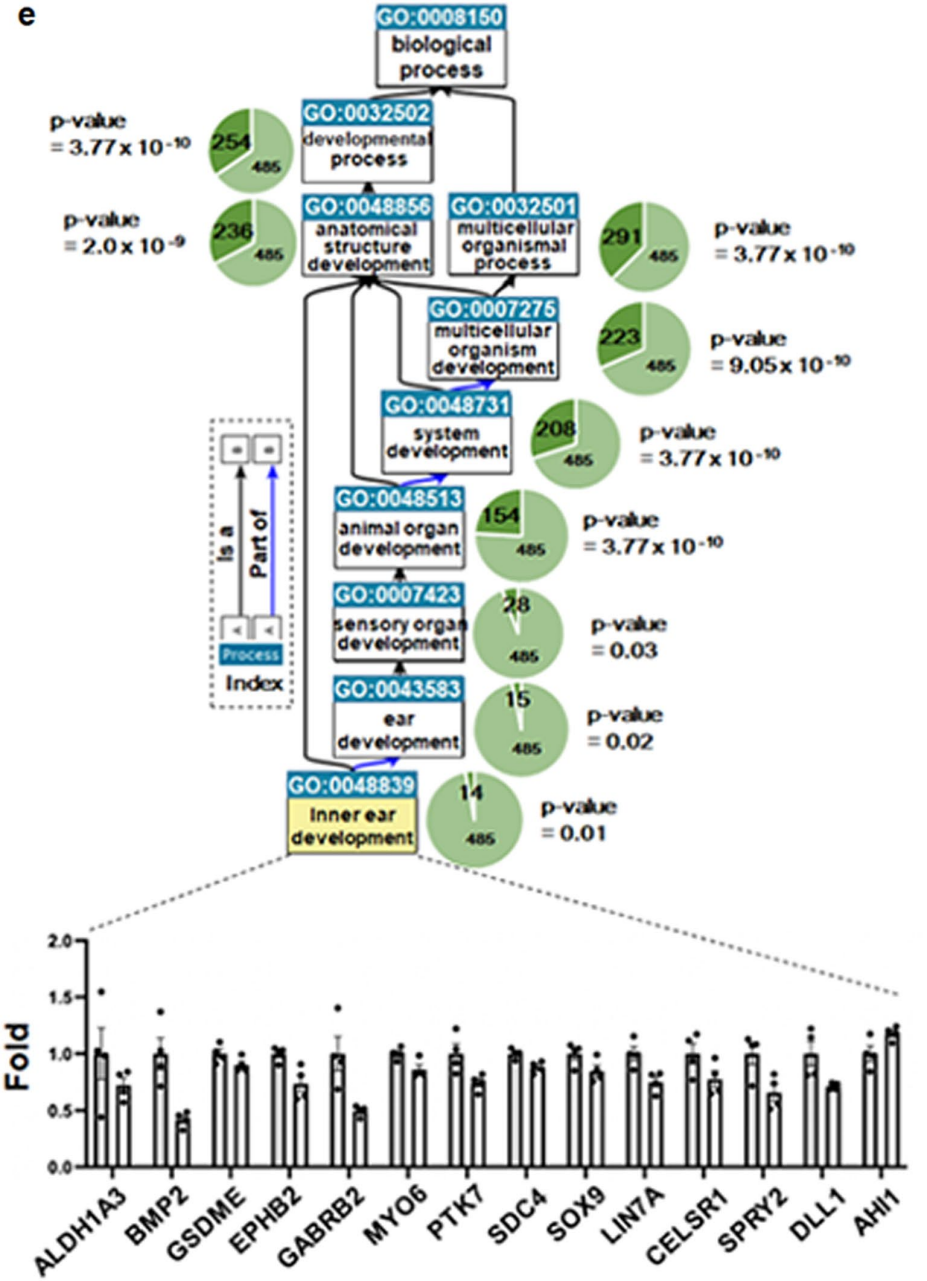
RNA sequencing analysis. (**a**) Schematic diagram of the analysis flow. (**b**) Volcano plot of significantly different genes (n = 630). Upregulation (red dot) and downregulation (blue dot) gene numbers were summarized as a pie graph (inlet). (**c**) Heatmap analyses of differential gene expression. The higher expression level was shown as red color while the lower expression was shown as blue. (**d**) (Upper) Revigo visualization of the top 30 gene ontology (GO) data. Clustered terms were listed in each box. (Bottom) Top 10 GO terms in biological process. The red dot box showed top2 GO terms, including cell differentiation and cellular developmental process. (**e**) Ancestor chart view of the QuickGO. In each GO term, enriched gene number was shown in the dark green pie while term size was shown as green pie with the adjust *p* value calculated by hypergeometric test and multiple testing correction (FDR). The colored arrow showed the relationship between the Ancestor term and the Child term. The black arrow showed “is a” and the blue arrow showed “part of”, which is explained in the index box. 14 genes which belong to the GO-term (GO: 0048839) were significantly enriched (adjusted *p* value = 0.01). 14 transcripts were significantly enriched for the GO term (GO: 0048839) (adjusted *p* value = 0.01). To verify their dysregulation, each FPKM value of 14 genes from the health group (gray, n = 4) and the hearing loss group (white, n = 4) was compared as fold change with standard error of the means (SEM). *p < 0.05 was the result of a statistical significance test using the Student's *t-*test (bottom).

We performed RNA sequencing analysis to investigate comprehensively the molecular pathways affected by *POU4F3* variants identified from the hearing-loss families. We used patient-derived lymphoblastoid cell lines (LCLs) to mimic the molecular regulatory programs of the original pathogenic circumstances, which were altered, at least in part, by these mutations. Figure 7a depicts the experiment and analysis flow. For subsequent sequencing analysis, we read over 100,000,000 reads from eight RNA-sequencing libraries. Around 95% of the reads passed quality inspection (Q > 30), and an average of 95% reads were successfully mapped to the human reference genome (GRCh37), covering approximately 20,000 human genes (Table S1).

Prior to the transcriptome analyses, we performed a correlation analysis to determine whether these cells exhibited the molecular features of the cochlea. We used mouse transcriptome data, including adult cochlear inner and outer hair cells, and early postnatal cochlea, because public RNA-sequencing data for human cochlear tissues were unavailable. We used adult mouse testis transcriptome data as negative controls. Table S2 summarizes the public data used in this study. The Spearman's correlation coefficients ranged from 0. 4 to 1, indicating a positive correlation (Fig. S5a,b). Statistically significant *p* values were found in two reference sets: 0.0399 for postnatal day 4 cochlea transcriptomes with patient samples and 0.0404 for postnatal day 7 cochlea transcriptome with patient samples (Fig. S5a,c). There is a moderate correlation between the reference transcriptome and the patient cell transcriptome in each case, as shown by the Spearman's correlation coefficients of 0.53 and 0.521. Of note that both postnatal day 4, and postnatal day 7 cochlear transcriptome have a slightly stronger correlation with adult cochlear transcriptome with an average coefficient of roughly 0.75, suggesting that these transcriptome favorably share similar molecular pathways. However, there was no correlation between the sample transcriptome, adult testis transcriptome, and the other reference transcriptome (Fig. S5). Overall, our RNA-seq data would reflect more of the molecular signature of the early postnatal cochlea (postnatal day 4 and day 7) rather than other response pathways reported in adult tissues.

After observing that RNA-seq data contain more early postnatal cochlear regulatory molecular pathways, we analyzed differential gene expression between wild-type and hearing-loss groups. 630 genes had statistically significant expression variations, which were represented using a volcano plot and a heatmap split into upregulated (n = 203) and downregulated (n = 427) groups (Fig. 7b,c). We used GO (Gene Ontology) analysis to elucidate the biological processes underpinning the observed dysregulation. Approximately 360 GO terms associated with biological processes were substantially enhanced. Revigo was used to further visualize the top 30 GOterms, displaying them as representative. The most distinguishable groups consisted of cellular differentiation, cellular developmental processes, and other GO keywords pertaining to development (Fig. 7d). This trend was also seen in the top ten GO-terms. Two of the most enriched GO-terms (*p* = 1.18E−11 and 1.8E−11, respectively) were cell differentiation (GO:0030154) and cellular developmental processes (GO:0048869) (Fig. 7d). We further enhanced our studies by showing an ancestor-child view of the enriched GO terms (Fig. 7e). Developmental process is an ancestor term, and inner ear developmental GO-term is its child term (GO:0048839). Noteworthy is the fact that 14 genes are differentially expressed in GO:0048839 in the hearing loss group, with a *p* value of 0.01. Its other ancestor GO-term is either ear development (GO: 0043583) or anatomical structure development (GO:0048856), for which 15 or 236 genes are enriched in each term, respectively. Using this ancestor chart, we determined that inner ear development is the most enriched child GO-term in the hearing-loss population. We next examined the differential expression of the 14 genes in the inner ear developmental GO-term (Fig. 7e, bottom bar graph). In comparison to the control group, the hearing-loss group demonstrated repressive patterns.

To determine if these genes and *POU4F3* are physically and functionally associated, we performed STRING analyses under the assumption that mRNA expression level is linearly correlated with translation. We observed possible protein associations in three groups (Fig. S6). Notable was the association of 14 dysregulated genes with the Notch pathway (DLL1), BMP pathway (BMP2), and Wnt (SDC4, PTK7, and CELSR1) pathway, which are connected to Sox9. These pathways have been reported as critical regulators to produce hair cells and dysregulations of them could lead to the hearing loss^14^. Initially, misregulation of BMP pathway was validated by measuring BMP2 expression level quantitatively (Fig. S7).

Interestingly, *POU4F3* clustered together with *MYO6* and *DFNA5*, mutations or dysregulations of which have been found in hearing loss studies^1,15–17^. Since we observed *MYO6* expression significantly dysregulated in the hearing-loss group, we further validated by RT-qPCR. Even though marginal, quantitative analyses reached statistical significance, suggesting the *POU4F3* variant regulates *MYO6* expression in the hearing loss pathology (Fig. S7). In the case of *AHI1*, even though we did not see the association in the STRING analyses, we further confirmed its expression level by RT-qPCR (Fig. S7) since it has been associated with non-syndromic deafness^18^. Overall, we confirmed dysregulation of *BMP2*, *MYO6* and *AHI1* by RT-qPCR. We concluded that the *POU4F3* variations might regulate Wnt, Notch, and/or BMP pathways, specifically leading to the misregulation of *BMP2*, *MYO6*, and *AHI1* in the pathogenesis of hearing loss.

In addition to the 14 enriched genes involved in the development of the inner ear, we also analyzed known *POU4F3* target genes, including *Lhx3, Gfi1, Bdnf, Ntf3, MYO6, Caprin1, and Nr2f2*. The majority of these genes, with the exception of *Lhx3* and *Ntf3*, were expressed based on transcriptome analysis, with average read-counts of 6224.024 and an average normalized value of 677.85 (Fig. S8a). Statistically significant downregulation was seen in *Bdnf* and *MYO6* in the hearing loss group, compared to the hearing loss group. The expression level of *Bdnf* was repressed by 93 percent, representing the most significant reduction. A reduced pattern was observed except for *Gfi1* and *Caprin1*, but the *p* value exceeded 0.05 (Fig. S8). Overall, the dysregulation of *POU4F3* downstream targets was reaffirmed.

After that, we explored whether the four variants had distinct transcriptome signatures. Spearman’s correlation analysis revealed that SB218 was less correlated to the other three cases (Fig. S9a). Notably, the SB218 variant (p.Ala189SerfsTer26) altered the subcellular location. DEseq2 was used to pinpoint the dysregulation between the SB218 variant and the others. We used fold-changes and expression levels following the visualization of the MA-plot (Fig. S9b). These patterns of expression clearly demonstrated upregulation or downregulation (Fig. S9c). Then, we studied the enriched biological processes to observe numerous categories, including cellular processes, synthesis, stimuli responses, cellular process regulation, metabolic process regulation, and cellular localization (Fig. S9d). Nuclear import (GO: 0051170) was one of the significantly enriched GO-terms (Fig. S9e). We narrowed them down further to 51 differentially expressed genes. In general, the SB218 variant (p.Ala189SerfsTer26) exhibited a diminished pattern (Fig. S9f). This further confirmed that the SB218 variant (p.Ala189SerfsTer26) dysregulated the nuclear import process at a molecular level.

## Discussion

POU-specific and POU-homeodomain regions of POU transcription factors have high-affinity DNA binding^19,20^. The presence of novel *POU4F3* variants in key regions encoding DNA-binding sites suggests that mutant proteins may have reduced ability to bind to DNA targets, resulting in insufficient target gene expression. Consistent with previous studies^2,3^, Protein modeling and structure analysis reveal that missense variants disrupt the interhelical interface of DNA-binding functional domains, reducing protein expression and stability. Weaker bands of the mutant proteins are detected in western blot analysis due to greater instability compared to wild-type protein, as confirmed by the CHX chase assay. As such, tertiary protein structure changes in the interhelical interface caused by *POU4F3* variants may affect DNA-binding ability, leading to reduced transcriptional activity for regulating downstream target gene expression related to inner ear hair cell function. Resultantly, we identified a significant reduction of the transcriptional activity necessary to induce regulation of downstream target gene expression.

Several downstream targets of *POU4F3*, including *Lhx3*^21^, *Gfi1*^22^, *Bdnf*^23^, *Ntf3*^23^, *MYO6*^24^, *Caprin1*^25^, and *Nr2f2*^26^, were known to regulate inner ear hair cell functions. We further identified altered expression of 14 downstream target genes associated with inner ear development using patient-derived LCLs. The alteration of genes that affect inner ear development could cause not infrequently only subclinical and silent phenotype until the accumulated effect inherent from the developmental defect leads to an obvious hearing loss later in adulthood. Unless the developmental sequela caused by the gene is very severe, the cumulative effect of the sequela can manifest the phenotype only after a certain point in such deafness cases. Enlarged vestibular aqueducts, one of the most common inner ear developmental anomalies, showed varying degrees of progressive hearing loss frequently involving their adulthood and even mid ages. Specifically, we observed that *POU4F3* variants downregulated the expression of *MYO6*, essential for maintenance of stereocilia of the hair cells, which is responsible for auditory mechanoelectrical transduction^24^. The altered expression of downstream *POU4F3* targets identified herein may support the a mechanistic basis for hearing loss caused by *POU4F3* variants. First, PCR and Sanger sequencing confirmed that *POU4F3* was obviously expressed in both HEK293T and patient-derived LCLs (Fig. S10), suggesting that these cell lines could at least provide the transcriptional environment to assess *POU4F3* function. Next, the downstream targets (*Gfi1*, *Bdnf*, *MYO6*, *Caprin1*, and *Nr2f2*) of *POU4F3* was identified to be expressed in the LCLs, and some of them were misregulated. Further, *POU4F3* was connected functionally and physically with 14 mis-regulated genes identified in the LCLs. More specifically, the Notch, Wnt, and BMP pathways associated with 14 dysregulated genes were functionally linked with *Myo6* and *DFNA5*, which clustered together with *POU4F3*. Finally, but not least, there was a significant moderate correlation of the expression profile between patient-derived cells and the cochlear hair cells. Collectively, the results provide evidence that patient-derived LCLs can be implicated in the transcriptome study of genetic hearing loss for studying *POU4F3* transcriptional function.

In this study, *POU4F3* variants were associated with distinct subcellular localization patterns. The POU homeodomain contains two putative NLSs required for proper *POU4F3* trafficking into the nucleus: N-terminal monopartite (amino acids 274–278; RKRKR) and C-terminal bipartite NLS (amino acids 314–331; KKNVVRVWFCNLQRQKQKR). Weiss et al.^2^ demonstrated that variants affecting monopartite NLS, bipartite NLS, or both, exerted differential effects on abnormal subcellular localization during the nuclear import process. As such, it is conceivable that the frameshift variant producing a truncated protein (p.Ala189SerfsTer26), lacking both the mono- and bi-partite NLSs, localized exclusively in the cytoplasm. Weiss et al.^2^ previously reported that a truncated *POU4F3* protein lacking both the mono- and bi-partite NLSs exhibited significantly aberrant localization in the cytoplasm (˃ 80%), compared to other truncated *POU4F3* proteins lacking only the monopartite (approximately 23%) or the bipartite NLSs (approximately 47%). This suggests that both NLSs contribute significantly to nuclear trafficking of *POU4F3*^2^. In addition, we observed differentially expressed transcripts associated with cellular localization between p.Ala189SerfsTer26 and three other missense variants, suggesting that aberrant nuclear import affects the ability to activate downstream targets. Among 51 DEGs related to nuclear import, expression of Frizzled-7 receptor (FZD7) was the most downregulated. Specifically, FZD7 interacts with the Wnt/β-catenin pathway^27,28^, which is required for cochlear hair cell differentiation^29^. Thus, the truncated *POU4F3* protein lacking both the mono- and bi-partite NLSs led to the downregulation of FZD7 expression, which might reduce nuclear β-catenin accumulation and, in turn, possibly affect cochlear air cell differentiation. Of note that these genes are expressed in our RNA-seq. data, further supporting that experimental design and conclusion reflect the hearing loss pathology.

Interestingly, western blot analysis showed that the expression of the mutant protein (p.Ala189SerfsTer26) was stronger than the wild-type protein. Moreover, p.Ala189SerfsTer26 mutant was more stable than the wild-type protein upon protein stability assays. This was in agreement with a previous study, which demonstrated that a mutant *POU4F3* protein (p.Ile295Thrfs*5) was more stable than the wild-type protein^2^. It has also been demonstrated that the mutant protein had a significantly greater half-life than the wild-type protein^2^. Although the exact mechanism remains poorly understood, recent studies have demonstrated that the ubiquitin–proteasome system is interrelated with the bipartate NLS function with regard to regulation of protein stability^11,30^. The cellular protein quality control system (i.e., ubiquitin–proteasome system) regulates the half-lives of various regulatory proteins and removes misfolded proteins^31^. NLS defects, including those of lysine ubiquitination sites, could decrease protein degradation while upregulating their half-lives^30^. In this sense, the p.Ala189SerfsTer26 mutant lacking the bipartite NLS (including lysine ubiquitination sites) would be likely to be more stable than the wild-type protein, similar to the extended half-life of the mutant protein (p.Ile295Thrfs*5). However, this variant might undergo nonsense-mediated mRNA decay in vivo, in contrast with the in vitro situation.

In this study, the predominantly nuclear localization of the p.Val318Met-*POU4F3*, residing within a bipartite NLS, did not perfectly align with the classical hypothesis. Lin et al.^11^ demonstrated that the missense variant (p.Lys328Glu), in which bipartite NLS amino acid residues were affected, was associated with aberrant *POU4F3* subcellular localization, which was in contrast with the present study. It has been suggested that the substitution of a basic lysine with an acidic glutamate (p.Lys328Glu) may alter bipartite NLS molecular properties, highlighting the importance of correct basic amino acid cluster alignment of bipartite NLS in maintaining the *POU4F3* protein localization.^11^ Indeed, bipartite NLS basic amino acids, the first two (KK) and the last two (KR), are considered essential for nuclear localization of *POU4F3*.^2^ The missense variant in this study (p.Val318Met), with conserved bipartite NLS basic amino acids and molecular properties, may therefore show normal nuclear localization, albeit with disrupted hydrophobic interactions.

At this point, the exact mechanism explaining why the NSHL phenotype in affected individuals carrying p.Val318Met is more severe than in those carrying p.Ala189SerfsTer26 remains unclear. Although the p.Ala189SerfsTer26 mutant, which lacks the bipartite NLS, fails to localize to the nucleus, it appears to be more stable than the wild-type protein. Alternatively, this mutant might undergo nonsense-mediated mRNA decay in vivo, leading to haploinsufficiency, which preserves one copy of the gene. On the other hand, the p.Val318Met mutant exhibits normal nuclear localization but has structural instability. If the molecular function of this mutant results in a dominant-negative effect, the NSHL phenotype would be more severe in cases with haploinsufficiency. However, phenotypic variability cannot be solely explained by different genotypes and pathogenic molecular mechanisms. Phenotypic variability in terms of age of onset, degree of severity, and configuration has been documented in previous studies^7–13^. Moreover, intrafamilial variations in age of onset and severity of hearing loss have been observed. Additionally, the phenotypic variability and the lack of a definitive genotype–phenotype correlation in DFNA15 might be attributable to a combination of stochastic and epigenetic factors. As such, our study's findings demonstrate that patient-derived LCLs can be effectively utilized in transcriptome studies of genetic hearing loss to investigate *POU4F3* transcriptional function. This approach offers a breakthrough for cases where obtaining human cochlear samples for transcriptome studies is not feasible. Nevertheless, it is important to note that *POU4F3*-expressing inner ear hair cells, such as iPSC-differentiated hair cell-like cells and otic organoids, could potentially provide further insights into the molecular mechanisms underlying *POU4F3*-associated DFNA15 and phenotype-genotype correlations. In addition, it is essential to move beyond studying the single gene's individual function and instead examine its broader contribution within the molecular networks. Our current knowledge of its full range of binding partners and regulatory elements remains incomplete. Therefore, conducting systemic and network analyses would be necessary to further elucidate the functions and interactions underlying *POU4F3*-associated DFNA15.

## Materials and methods

### Subjects

All procedures in this study were approved by the Institutional Review Boards of Seoul National University Hospital (IRB-H-0905-041-281) and Seoul National University Bundang Hospital (IRB-B-1007-105-402). Written informed consent was obtained from both affected and unaffected members of the families. In the case of pediatric participants, written informed consent was obtained from their parents or guardians. All methods were performed in accordance with the relevant guidelines and regulations. The affected individuals underwent comprehensive phenotypic evaluations, including medical history interviews, physical examinations, imaging, and audiological and vestibular assessments.

### Audiological assessments

Pure tone audiometry (PTA) with thresholds of air and bone conduction was performed in all the probands, as well as the affected individuals if possible, according to the standard protocols. The hearing thresholds for six different octaves (0.25, 0.5, 1, 2, 4, and 8 kHz) were evaluated using PTA. The frequency ranges were classified as follows: low frequency, 250–500 Hz; mid-frequency, 1–2 kHz; and high frequency, 4–8 kHz. The mean hearing threshold was calculated as the mean of the thresholds at 0.5, 1, 2, and 4 kHz, and the severity of hearing loss was classified as mild (20–40 dB), moderate (41–55 dB), moderately severe (56–70 dB), severe (71–90 dB), or profound (> 90 dB), as previously reported. The audiogram configurations were classified as down-sloping, rising, or flat. Specifically, the down-sloping configuration on PTA was defined by increasing the thresholds from 0.25 to 8 kHz, while the difference of the thresholds between 0.25 and 8 kHz was > 20 dB HL^32^.

### Molecular genetic testing

Genomic DNA was extracted from peripheral blood using the standard procedure and was subjected to exome sequencing using a Sure Select 50 Mb Hybridization and Capture Kit and a HiSeq2000 platform in four proband samples. The paired-end read length was 100 bp, and the reads were aligned using the University of California Santa Cruz (UCSC) hg19 reference genome browser (https://genome.ucsc.edu/). As described previously,^33,34^ bioinformatics analysis and strict filtering were performed to retrieve candidate variants of hearing loss. The candidate variants were confirmed through Sanger sequencing, and a segregation study was performed using parental DNA samples whenever available. The pathogenicity of the four novel *POU4F3* variants was classified using the ACMG/AMP guidelines for hearing loss.^35,36^ The in-silico prediction analysis for candidate variants in each family are presented in Table S3.

### Structural analysis of *POU4F3*

AlphaFold Protein Structure Database generated the model structure of *POU4F3*^37,38^. POU homeodomain and POU-specific domain were assembled with the DNA binding cleft in between. The homeobox protein HOX-B1/DNA ternary complex (PDB ID: 1B72) was aligned to the *POU4F3* structure to allow DNA-binding analysis^39^. The stabilities of truncated *POU4F3* variants were evaluated based on the PAE score, which reflects the inter-domain accuracy. All the figures were generated using PyMOL (ver. 2.5.2) software (PyMOL Molecular Graphics System ver. 2.0, Schrödinger Inc., New York, NY, USA).

### Molecular study

The series of steps for the molecular work, including plasmid construction, cell culture, transfection, western blot, immunocytochemistry, real-time quantitative reverse transcription-polymerized chain reaction, and luciferase assay, are all described in Table S4.

### RNA sequencing and bioinformatic analysis

The detailed information of RNA sequencing and bioinformatic analysis is described in Table S5.

### Statistics

The detailed information of statistics conducted in this study is described in each Figure legend.

### Supplementary Information

Supplementary Figures.

Supplementary Information.

## Data Availability

Sequence variations were submitted to Clinvar with the accession code (SCV003926516, SCV003926517, SCV003926518, SCV003926519). The authors confirm that the data supporting the findings of this study are available within the article and its supplementary materials. The data (Individual-level whole-exome sequence data and RNA-sequencing data) that support the findings of this study are available from the corresponding author (B.Y.C., choiby2010@gmail.com) upon reasonable request. Some data may not be made available because of privacy or ethical restrictions.
