# Supplementary material for: Ramifications of POU4F3 variants associated with autosomal dominant hearing loss in various molecular aspects
Source: Sci Rep. 2023 Aug 3;13:12584. doi: 10.1038/s41598-023-38272-w (PMC10400627; doi:10.1038/s41598-023-38272-w)
Supplement: Supplementary file 1 — Supplementary Figures. [file 41598_2023_38272_MOESM1_ESM.pdf]

Ramifications of *POU4F3* variants associated with autosomal dominant hearing loss in various molecular aspects

Sang-Yeon Lee, Min Young Kim, Jin Hee Han, Sang Soo Park, Yejin Yun, Seung-Cheol Jee, Jae Joon Han, Jun Ho Lee, Heeyoung Seok*, Byung Yoon Choi*

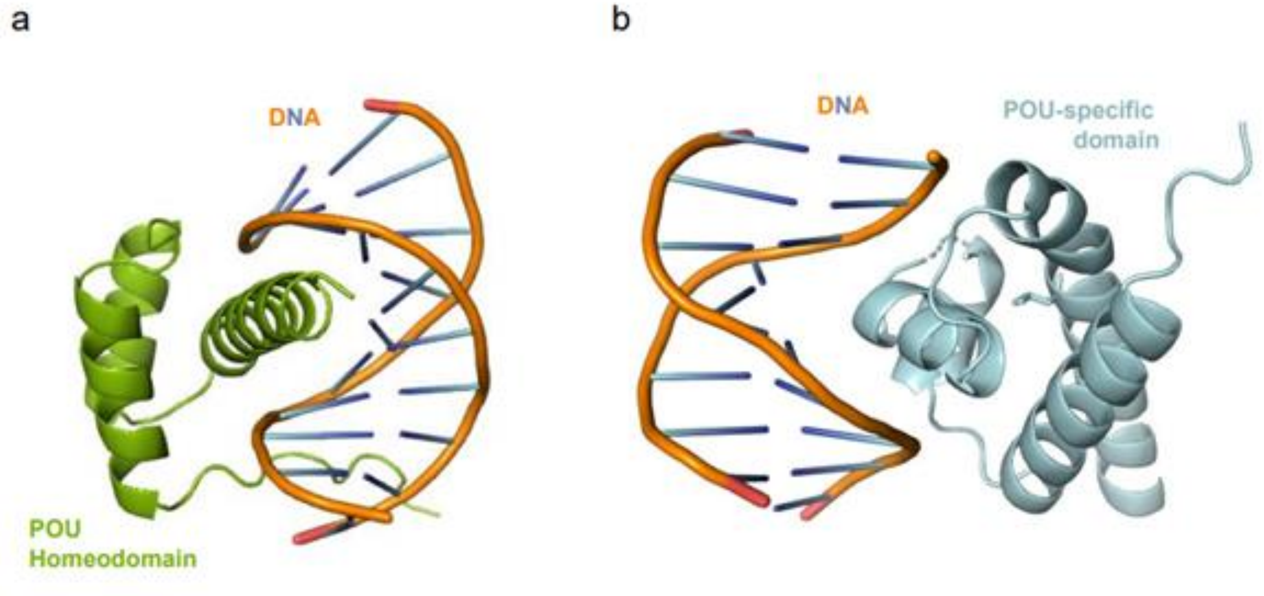

Fig S1. DNA-binding interface of Alphafold-generated POU4F3 model structure.
(a) POU homeodomain and (b) POU-specific domain. DNA-binding interface of POU4F3 was generated by 1cqt (OCT1) alignment.

Ramifications of *POU4F3* variants associated with autosomal dominant hearing loss in various molecular aspects

Sang-Yeon Lee, Min Young Kim, Jin Hee Han, Sang Soo Park, Yejin Yun, Seung-Cheol Jee, Jae Joon Han, Jun Ho Lee, Heeyoung Seok*, Byung Yoon Choi*

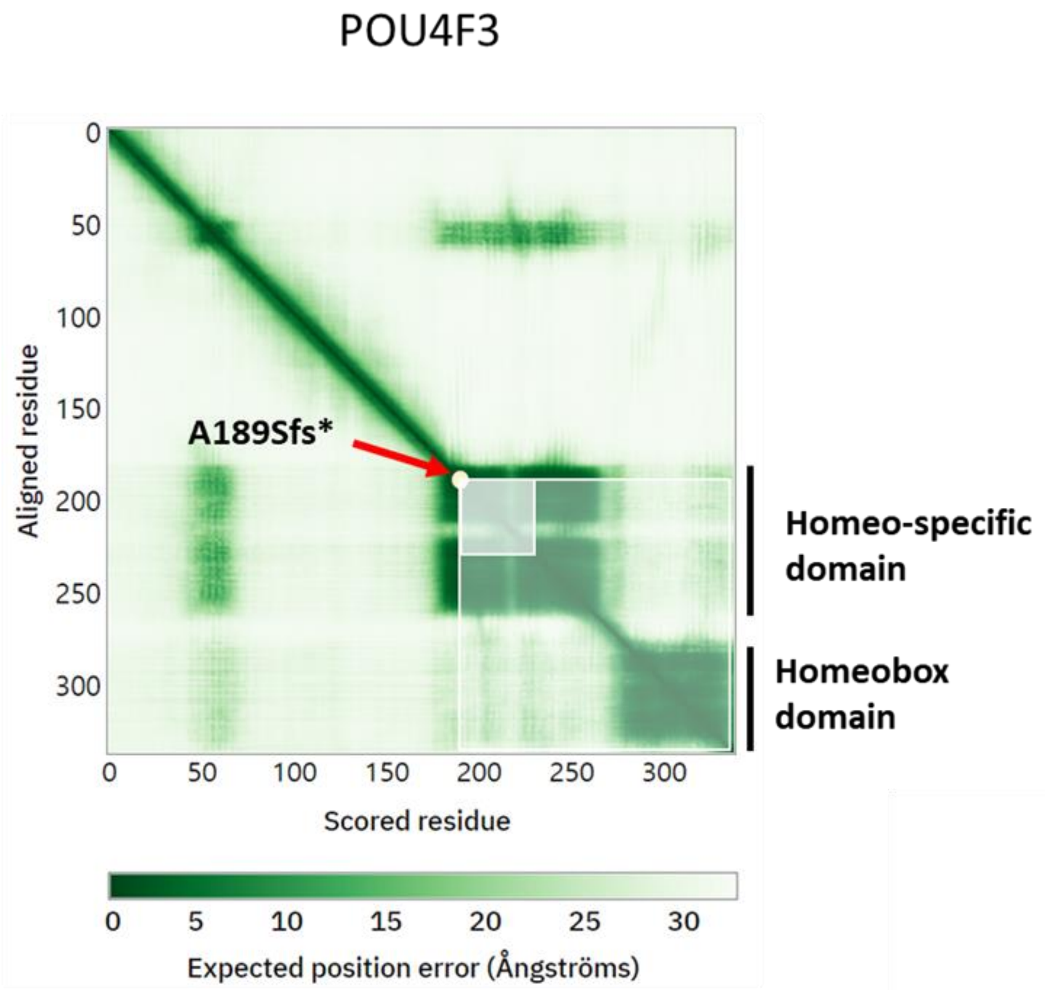

Fig S2. The Ala189Serfs*26 variant destabilized POU4F3 protein stability, as demonstrated by the predicted aligned error (PAE) score.

Ramifications of *POU4F3* variants associated with autosomal dominant hearing loss in various molecular aspects

Sang-Yeon Lee, Min Young Kim, Jin Hee Han, Sang Soo Park, Yejin Yun, Seung-Cheol Jee, Jae Joon Han, Jun Ho Lee, Heeyoung Seok*, Byung Yoon Choi*

a

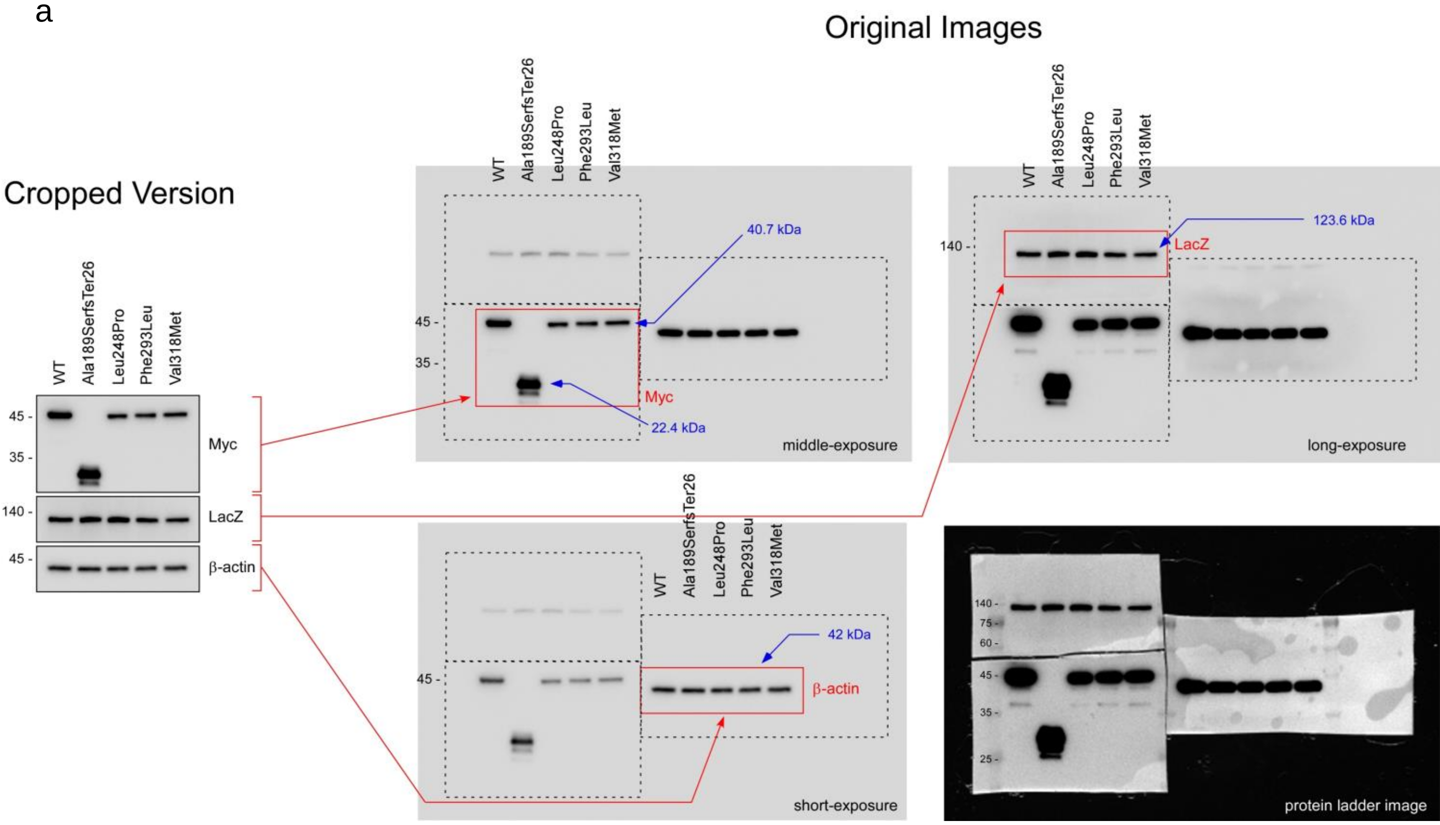

b

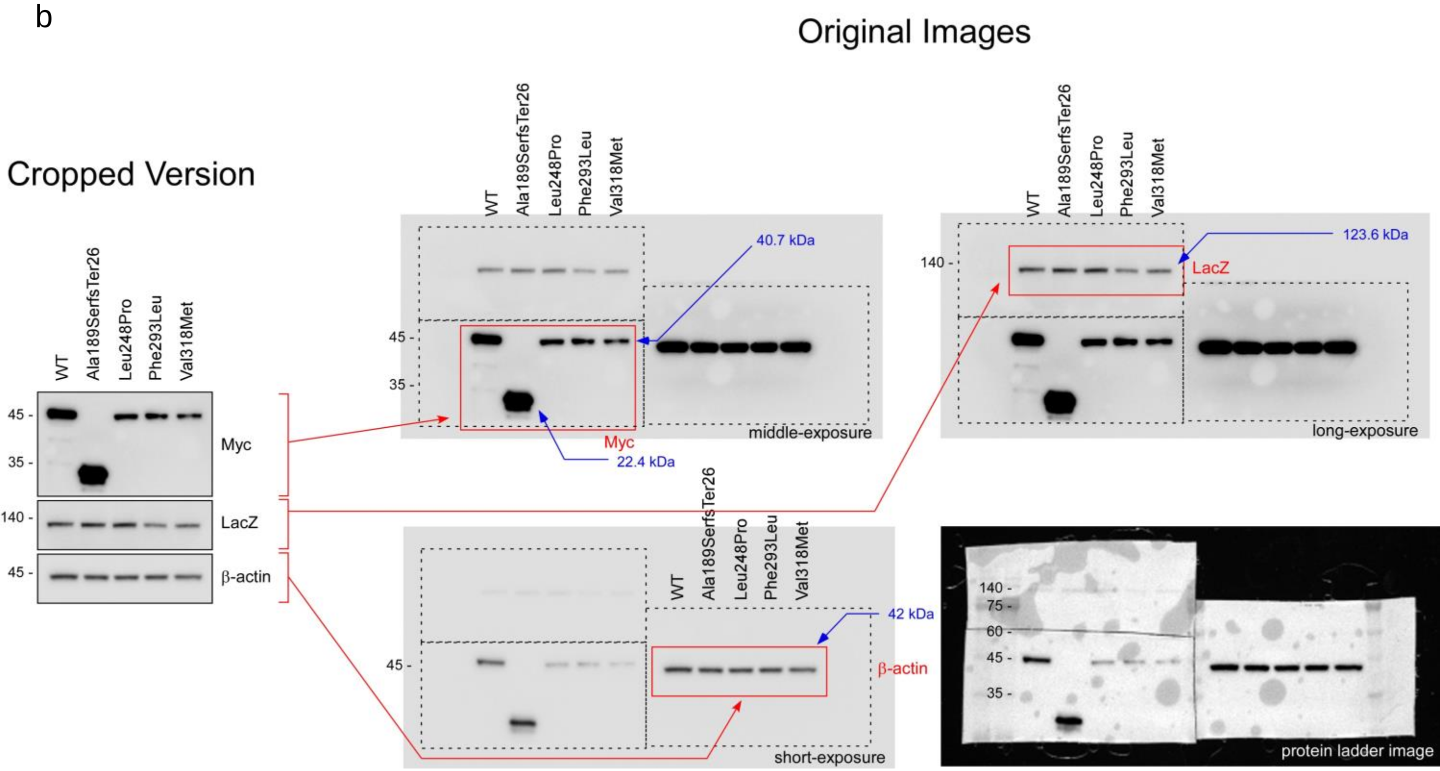

Fig S3. Original immunoblots for POU4F3 wild-type, frameshift, missense mutations by transient transfection at HEK293T cell. The immunoblots are representative of independent repetitive experiments. LacZ is used as a transfection control and b-actin is used as a loading control. Uncropped blot images for Fig 4a-c were shown in (a)-(c). (Continued on the next page.)

Ramifications of *POU4F3* variants associated with autosomal dominant hearing loss in various molecular aspects

Sang-Yeon Lee, Min Young Kim, Jin Hee Han, Sang Soo Park, Yejin Yun, Seung-Cheol Jee, Jae Joon Han, Jun Ho Lee, Heeyoung Seok*, Byung Yoon Choi*

c

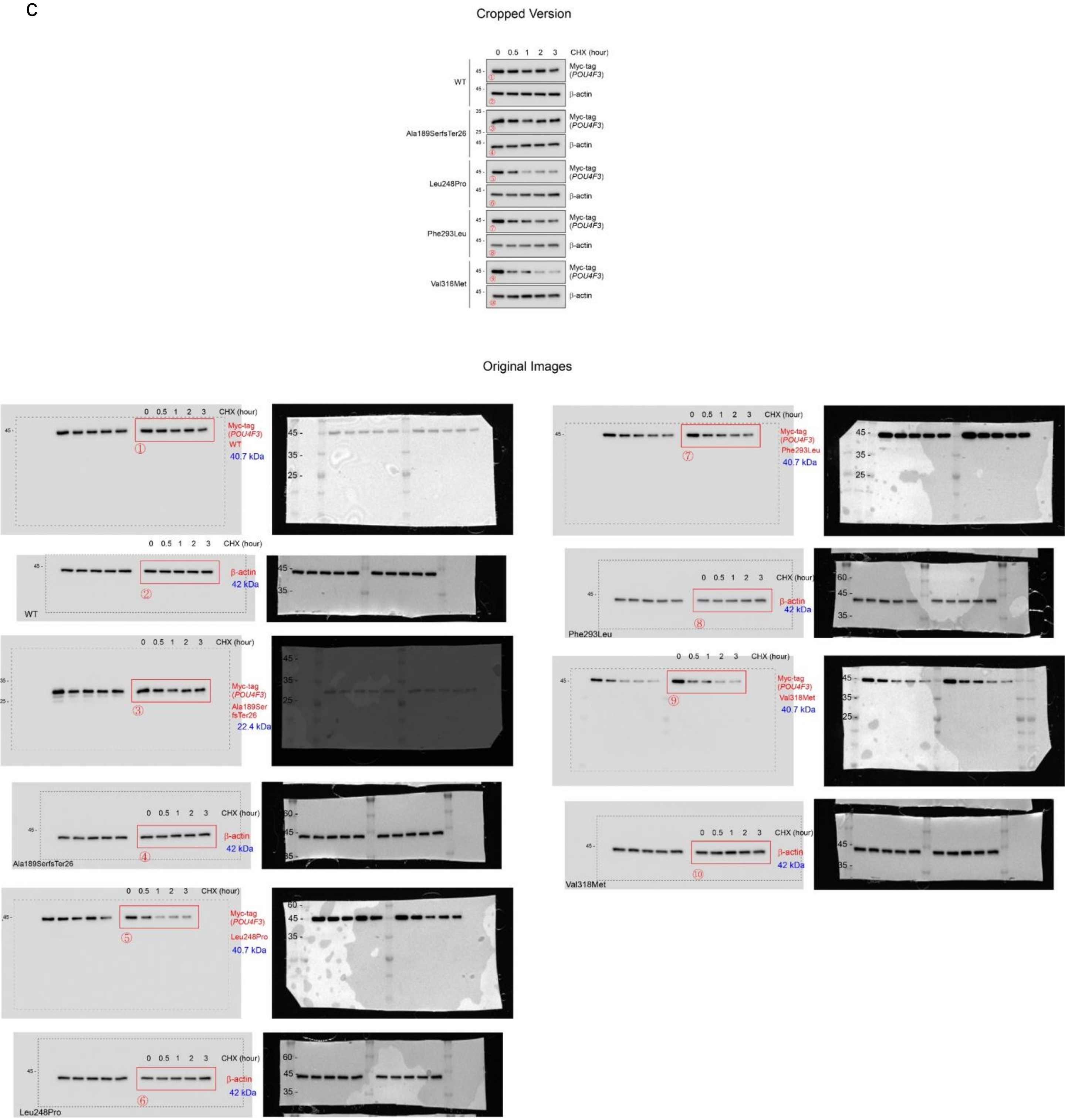

Fig S3. The original images corresponding to each cropped image are marked with red letters or arrows. The names of the samples in each lane are labeled the same as the cropped images. The standard protein size markers are indicated on the marker membrane and blot membrane in black text, and the expected molecular weight is indicated in blue text. Proteins present in each gel are indicated in red letters. The requested text is displayed so that it is not on top of the cropped image. Red boxes, edge of the cropped version images; Black dashed lines, edge of the membrane;

Ramifications of *POU4F3* variants associated with autosomal dominant hearing loss in various molecular aspects

Sang-Yeon Lee, Min Young Kim, Jin Hee Han, Sang Soo Park, Yejin Yun, Seung-Cheol Jee, Jae Joon Han, Jun Ho Lee, Heeyoung Seok*, Byung Yoon Choi*

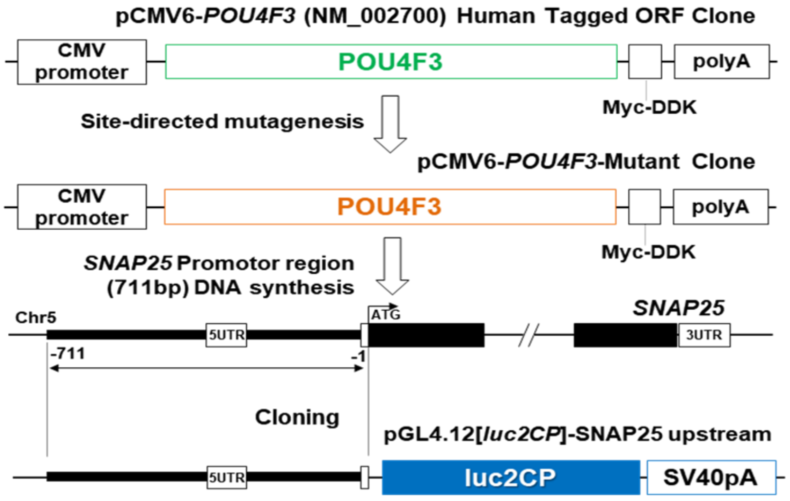

Fig S4. Schematic figure of cloning and site-directed mutagnesis for luciferase reporter assay. Vector information of luciferase assay and *POU4F3* pGL4.12 [luc2CP] vector features list and map are presented. After site-directed mutagenesis, the four variants in *POU4F3* expression plasmid were introduced with pCMV6-*POU4F3*-Mutant clone according to the manufacturer's protocol. A -711 bp upstream promoter region consisting of a mini-enhancer of the*SNAP25*, which is bound and transactivated by *POU4F3*, was inserted.

Ramifications of *POU4F3* variants associated with autosomal dominant hearing loss in various molecular aspects

Sang-Yeon Lee, Min Young Kim, Jin Hee Han, Sang Soo Park, Yejin Yun, Seung-Cheol Jee, Jae Joon Han, Jun Ho Lee, Heeyoung Seok*, Byung Yoon Choi*

a

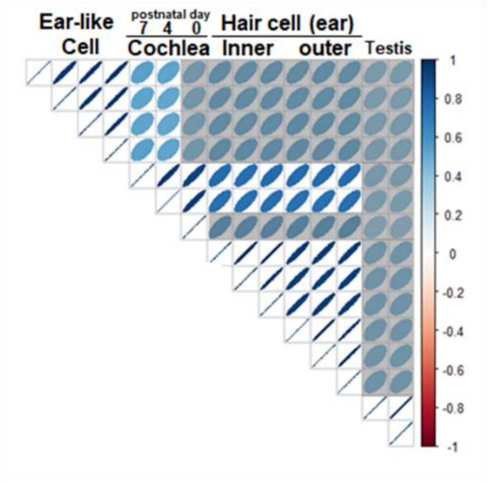

b

coefficient	h-earlike				Cochlea_p_7day	Cochlea_p_4day	Cochlea_p_0day	inner hair cell (adult)			outer hair cell (adult)			testis (adult)	
h-earlike	1.000	0.957	0.970	0.970	0.531	0.521	0.498	0.581	0.578	0.581	0.581	0.581	0.579	0.469	0.468
	0.957	1.000	0.969	0.971	0.529	0.519	0.496	0.586	0.582	0.587	0.585	0.586	0.584	0.466	0.466
	0.970	0.969	1.000	0.977	0.529	0.517	0.491	0.589	0.585	0.590	0.590	0.591	0.588	0.473	0.472
	0.970	0.971	0.977	1.000	0.535	0.524	0.498	0.595	0.592	0.596	0.595	0.595	0.593	0.477	0.476
Cochlea_p_7day	0.531	0.529	0.529	0.535	1.000	0.959	0.912	0.762	0.757	0.766	0.766	0.766	0.764	0.478	0.476
Cochlea_p_4day	0.521	0.519	0.517	0.524	0.959	1.000	0.945	0.747	0.742	0.751	0.747	0.748	0.745	0.469	0.467
Cochlea_p0_day	0.498	0.496	0.491	0.498	0.912	0.945	1.000	0.679	0.673	0.682	0.676	0.676	0.674	0.440	0.437
inner hair cell (adult)	0.581	0.586	0.589	0.595	0.762	0.747	0.679	1.000	0.984	0.992	0.946	0.945	0.944	0.519	0.519
	0.578	0.582	0.585	0.592	0.757	0.742	0.673	0.984	1.000	0.985	0.939	0.938	0.937	0.516	0.516
	0.581	0.587	0.590	0.596	0.766	0.751	0.682	0.992	0.985	1.000	0.947	0.947	0.945	0.519	0.519
outer hair cell (adult)	0.581	0.585	0.590	0.595	0.766	0.747	0.676	0.946	0.939	0.947	1.000	0.993	0.991	0.517	0.517
	0.581	0.586	0.591	0.595	0.766	0.748	0.676	0.945	0.938	0.947	0.993	1.000	0.992	0.517	0.516
	0.579	0.584	0.588	0.593	0.764	0.745	0.674	0.944	0.937	0.945	0.991	0.992	1.000	0.516	0.515
testis (adult)	0.469	0.466	0.473	0.477	0.478	0.469	0.440	0.519	0.516	0.519	0.517	0.517	0.516	1.000	0.997
	0.468	0.466	0.472	0.476	0.476	0.467	0.437	0.519	0.516	0.519	0.517	0.516	0.515	0.997	1.000

c

	h-earlike				Cochlea_p_7day	Cochlea_p_4day	Cochlea_p_0day	inner hair cell (adult)			outer hair cell (adult)			testis (adult)	
h-earlike	0	1.47E-15	4.85E-17	5.40E-17	0.041	0.041	0.059	0.150	0.151	0.148	0.153	0.154	0.152	0.115	0.115
	1.47E-15	0	3.41E-17	1.44E-17	0.042	0.042	0.059	0.167	0.169	0.164	0.170	0.171	0.169	0.110	0.110
	4.85E-17	3.41E-17	0	3.63E-19	0.038	0.037	0.052	0.167	0.169	0.164	0.170	0.172	0.170	0.119	0.119
	5.40E-17	1.44E-17	3.63E-19	0	0.040	0.039	0.054	0.171	0.172	0.168	0.174	0.175	0.173	0.115	0.115
Cochlea_p_7day	0.041	0.042	0.038	0.040	0	6.04E-14	5.03E-09	0.018	0.019	0.017	0.017	0.017	0.018	0.056	0.055
Cochlea_p_4day	0.041	0.042	0.037	0.039	6.04E-14	0	6.07E-11	0.031	0.032	0.029	0.031	0.031	0.031	0.059	0.058
Cochlea_p_0day	0.059	0.059	0.052	0.054	5.03E-09	6.07E-11	0	0.107	0.110	0.102	0.108	0.108	0.109	0.061	0.060
inner hair cell (adult)	0.150	0.167	0.167	0.171	0.018	0.031	0.107	0	1.39E-20	3.15E-24	1.81E-11	2.04E-11	2.23E-11	0.138	0.141
	0.151	0.169	0.169	0.172	0.019	0.032	0.110	1.39E-20	0	1.48E-20	3.75E-11	4.17E-11	4.50E-11	0.139	0.142
	0.148	0.164	0.164	0.168	0.017	0.029	0.102	3.15E-24	1.48E-20	0	1.58E-11	1.77E-11	1.95E-11	0.135	0.138
outer hair cell (adult)	0.153	0.170	0.170	0.174	0.017	0.031	0.108	1.81E-11	3.75E-11	1.58E-11	0	3.06E-25	7.34E-24	0.135	0.138
	0.154	0.171	0.172	0.175	0.017	0.031	0.108	2.04E-11	4.17E-11	1.77E-11	3.06E-25	0	2.61E-24	0.134	0.136
	0.152	0.169	0.170	0.173	0.018	0.031	0.109	2.23E-11	4.50E-11	1.95E-11	7.34E-24	2.61E-24	0	0.136	0.139
testis (adult)	0.115	0.110	0.119	0.115	0.056	0.059	0.061	0.138	0.139	0.135	0.135	0.134	0.136	0	1.03E-28
	0.115	0.110	0.119	0.115	0.055	0.058	0.060	0.141	0.142	0.138	0.138	0.136	0.139	1.03E-28	0

Fig S5. Correlation analyses of transcriptome between patient-derived cell lines and mouse models' RNA-sequencing data. (a) Spearman's correlation coefficient with statistical test results was visualized. A stronger positive correlation coefficient showed a linear shape with blue color. Shading indicates p-values greater than 0.05. (b) Spearman's correlation coefficients were shown as a table with higher values as red to lower as white. H-earlike sample is patient-drived transcriptome. Cochlea_p_7day = postnatal 7-day Cochlea; Cochlea_p_4day = postnatal 4-day Cochlea; Cochlea_p_0day = postnatal 0-day Cochlea. (c) p-value table from Spearman's correlation measurement. The red color showed a p-value lower than 0.05.

Ramifications of *POU4F3* variants associated with autosomal dominant hearing loss in various molecular aspects

Sang-Yeon Lee, Min Young Kim, Jin Hee Han, Sang Soo Park, Yejin Yun, Seung-Cheol Jee, Jae Joon Han, Jun Ho Lee, Heeyoung Seok*, Byung Yoon Choi*

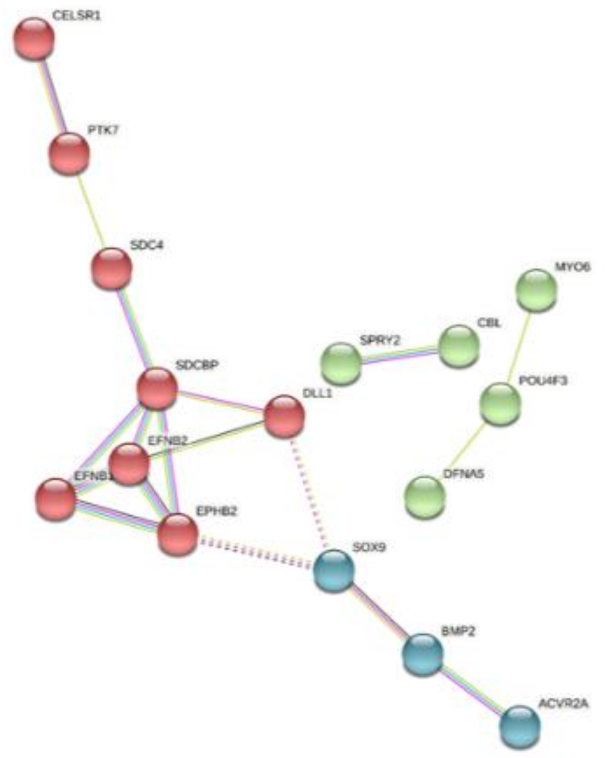

Fig S6. Functional and physical association of 14 enriched genes with POU4F3. 14 enriched genes and POU4F3 were clustered in 3 groups. The dotted line indicates the edge of the cluster. Three parallel lines showed interaction evidence. The thick line means strength of data support. Each colored line showed interactions. Sky blue line is known interaction with curated databases. The purple one is experimentally determined known interactions. Predicted interactions showed as green (gene neighborhood), red (gene fusion), and blue (gene co-occurrence). Another interaction is shown as either textmining (yellow-green) or co-expression (black).

Ramifications of *POU4F3* variants associated with autosomal dominant hearing loss in various molecular aspects

Sang-Yeon Lee, Min Young Kim, Jin Hee Han, Sang Soo Park, Yejin Yun, Seung-Cheol Jee, Jae Joon Han, Jun Ho Lee, Heeyoung Seok*, Byung Yoon Choi*

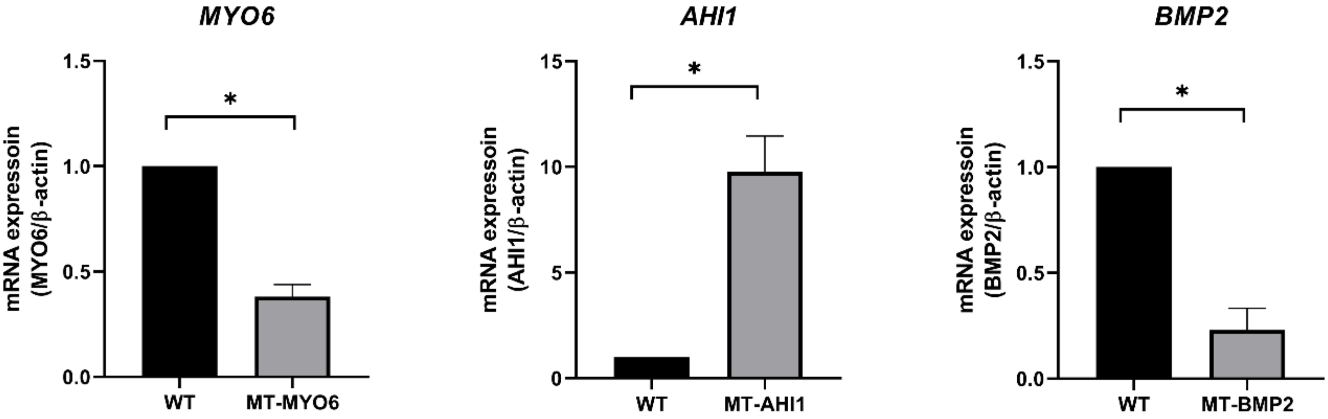

Fig S7. Validation of transcriptome analysis using RT-qPCR.

Ramifications of *POU4F3* variants associated with autosomal dominant hearing loss in various molecular aspects

Sang-Yeon Lee, Min Young Kim, Jin Hee Han, Sang Soo Park, Yejin Yun, Seung-Cheol Jee, Jae Joon Han, Jun Ho Lee, Heeyoung Seok*, Byung Yoon Choi*

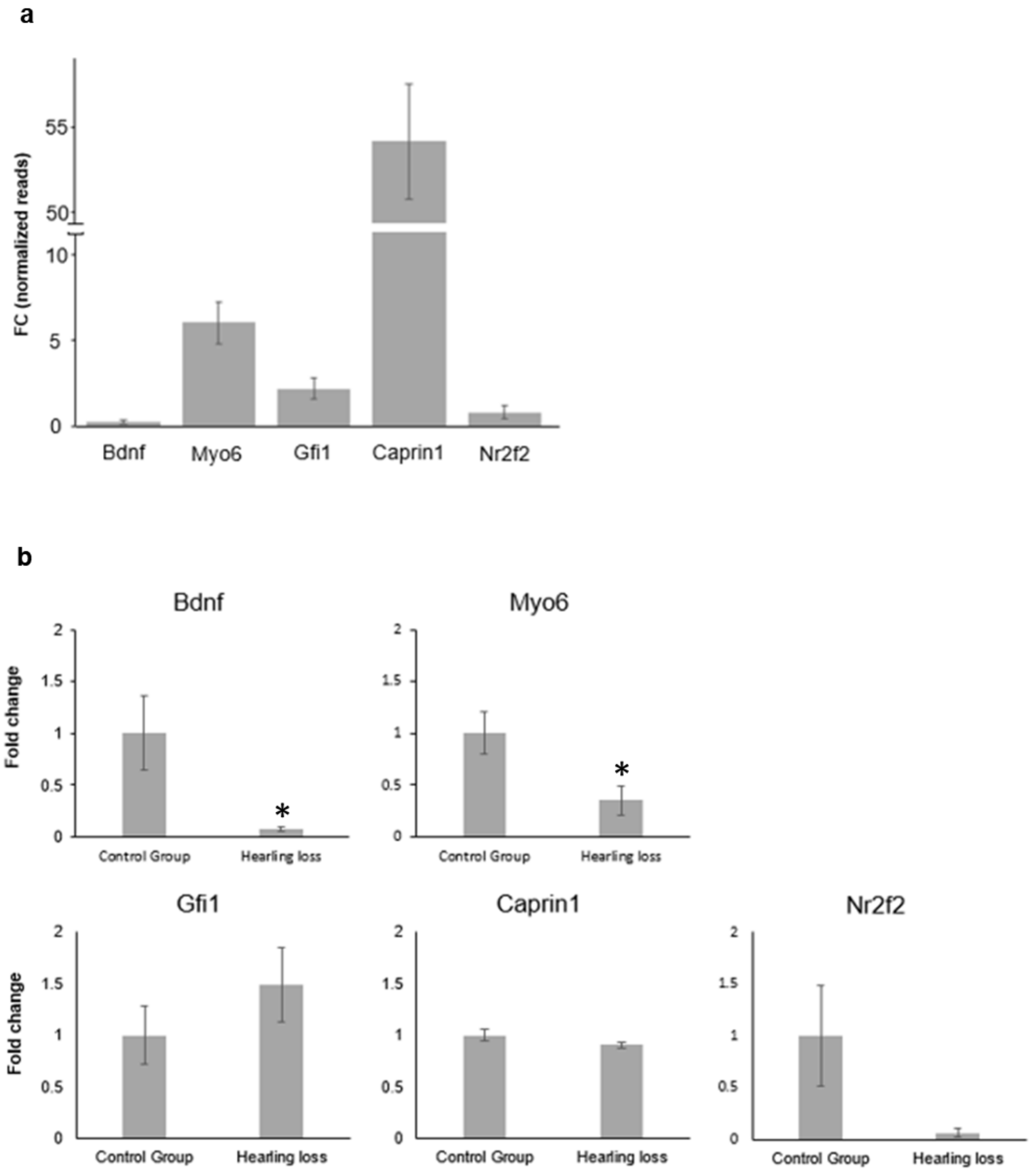

Fig S8. The expression level of known target genes. (a) Normalized reads showed expressional fold changes ranging from 1 to 55. (b) Bdnf and Myo6 showed statistically significant repression in the hearing-loss group (*; p-value <0.05). Gfi1, Caprin 1, and Nr2f2 showed their expression, but dysregulation was not significant.

Ramifications of *POU4F3* variants associated with autosomal dominant hearing loss in various molecular aspects

Sang-Yeon Lee, Min Young Kim, Jin Hee Han, Sang Soo Park, Yejin Yun, Seung-Cheol Jee, Jae Joon Han, Jun Ho Lee, Heeyoung Seok*, Byung Yoon Choi*

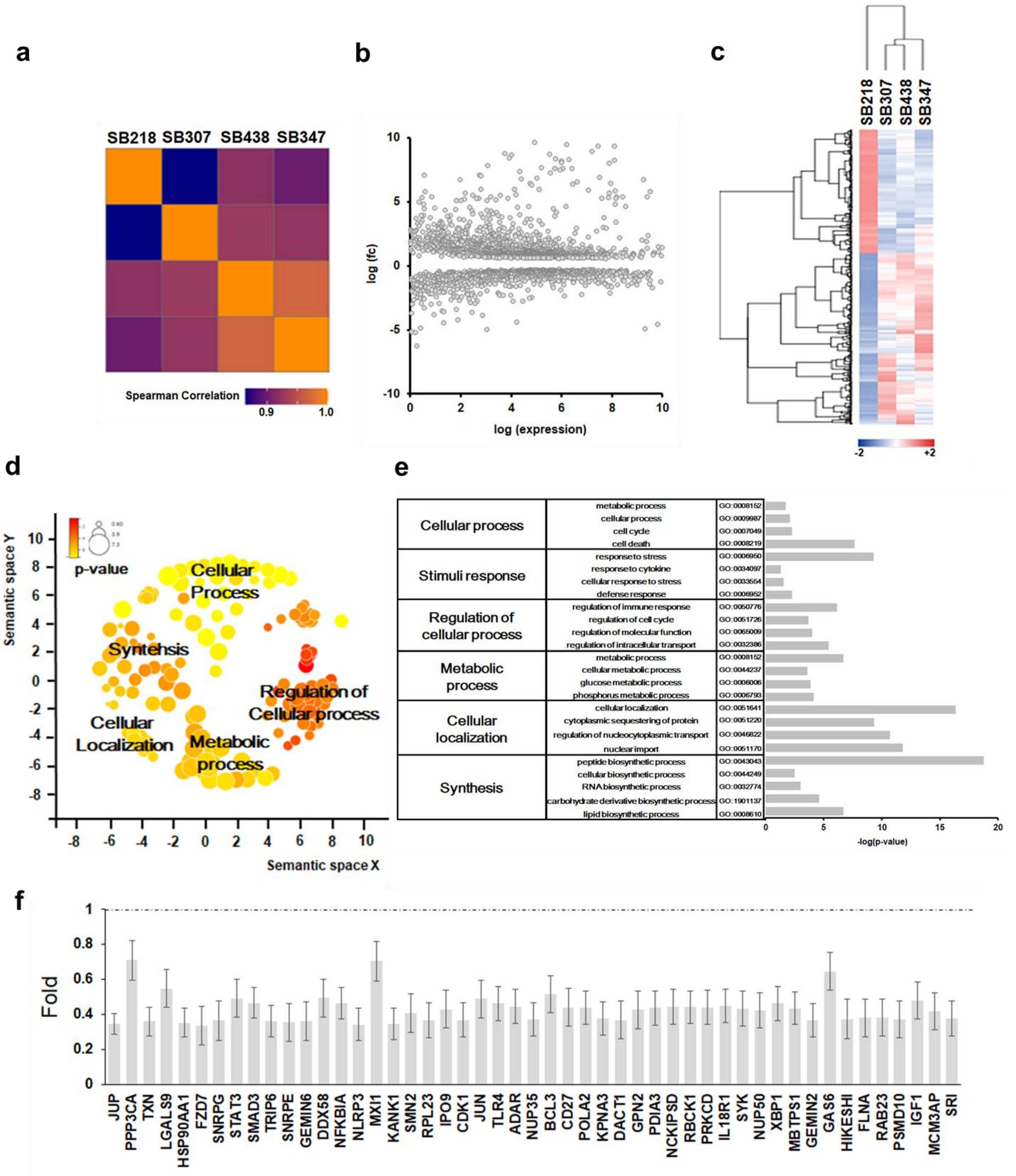

Fig S9. Transcriptome analyses (a) Spearman's correlation coefficient was shown as a heatmap. The yellowest is the highest expression. (b) MA-plot of the transcriptome of four patients. The x-axis indicates an individual's expression level, while the y-axis shows fold change. (c) Heatmap of the four patients' transcriptomes. The red color indicates higher expression. (d) Gene ontology (GO) analyses the listed genes under the Revigo visualization to show representative categories, including Cellular process, Synthesis, Cellular localization, Metabolic process, and regulation of the cellular process. (e) Representative lists of the significantly enriched GO terms in the biological process. (f) Gene expression fold-change among patients visualized as a bar graph in the nuclear import cluster.

Ramifications of *POU4F3* variants associated with autosomal dominant hearing loss in various molecular aspects

Sang-Yeon Lee, Min Young Kim, Jin Hee Han, Sang Soo Park, Yejin Yun, Seung-Cheol Jee, Jae Joon Han, Jun Ho Lee, Heeyoung Seok*, Byung Yoon Choi*

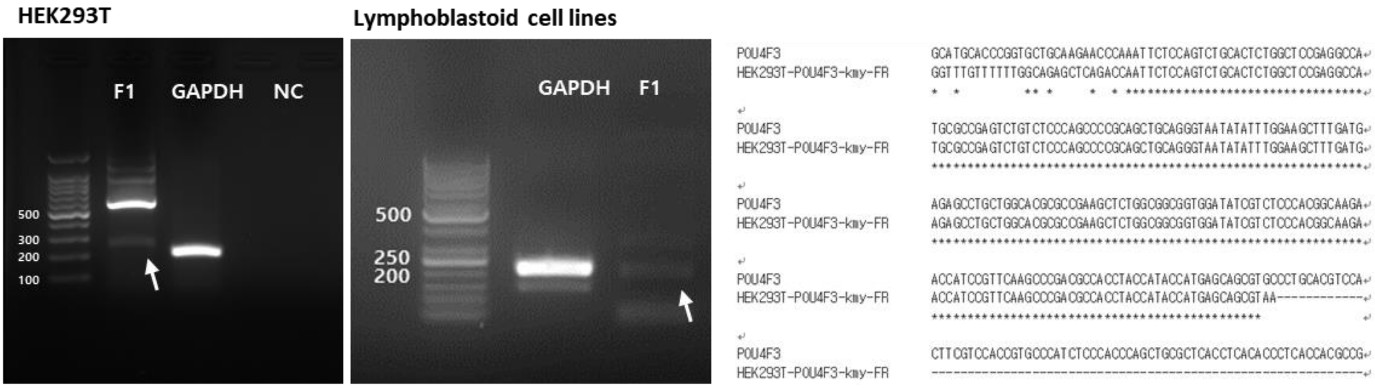

POU4F3-F1 (246bp): 5’-GAACTCCAAGCAGCCTTTCG-3’

POU4F3-R1: 5’-ACGCTGCTCATGGTATGGTA-3’

Fig S10. *POU4F3* expression in both human embryonic kidney cell line (HEK293T) and patient-derived lymphoblastoid cell lines.
