# Supplementary material for: Ramifications of POU4F3 variants associated with autosomal dominant hearing loss in various molecular aspects
Source: Sci Rep. 2023 Aug 3;13:12584. doi: 10.1038/s41598-023-38272-w (PMC10400627; doi:10.1038/s41598-023-38272-w)
Supplement: Supplementary file 2 — Supplementary Information. [file 41598_2023_38272_MOESM2_ESM.pdf]

Ramifications of *POU4F3* variants associated with autosomal dominant hearing loss in various molecular aspects

Sang-Yeon Lee, Min Young Kim, Jin Hee Han, Sang Soo Park, Yejin Yun, Seung-Cheol Jee, Jae Joon Han, Jun Ho Lee, Heeyoung Seok*, Byung Yoon Choi*

Table S1. Profiles of eight RNA-sequencing libraries

	**Total Reads**	**Reads (%, >Q30)**	**Processed Reads**	**Mapped Reads**
BHR (healthy control)	123,269,416	95.57	120,420,070	114,749,528
CBY (healthy control)	125,464,050	95.53	122,755,224	119,522,107
JHW (healthy control)	112,964,020	94.98	110,362,694	105,478,893
LSY (healthy control)	123,936,098	95.91	122,656,222	119,509,523
SB218 (hearing loss)	131,504,578	95.59	128,694,112	123,853,837
SB307 (hearing loss)	131,529,466	95.92	129,397,722	125,782,025
SB438 (hearing loss)	107,493,944	95.7	105,568,228	102,527,644
SB347 (hearing loss)	114,897,400	95.62	112,532,770	107,327,803

Table S2. A summary of the public data used in this study

SRR ID	Source Tissue	Organism	Age	Total Reads	Mapped Reads	Reference
SRR6798475	Cochlear inner hair cell	mouse	Adult	7,332,016	6,657,566	Yi Li et al, Scientific Data volume 5, Article number: 180199 (2018)
SRR6798476	Cochlear inner hair cell	mouse	Adult	229,368	2,085,153	
SRR6798477	Cochlear inner hair cell	mouse	Adult	12,585,587	11,397,869	
SRR6798479	Cochlear outer hair cell	mouse	Adult	14,468,752	12,296,204	
SRR6798481	Cochlear outer hair cell	mouse	Adult	12,215,142	10,377,025	
SRR6798482	Cochlear outer hair cell	mouse	Adult	7,161,499	6,087,056	
SRR1534779	Cochlea	mouse	Postnatal day 0	117,562,038	23,321,842	Scheffer D et al, The Journal of Neuroscience, April 22, 2015, 35(16):6366–6380
SRR1534787	Cochlea	mouse	Postnatal day 4	29,941,458	20,392,791	
SRR1534792	Cochlea	mouse	Postnatal day 7	36,343,531	23,813,133	
SRR15597783	Testis	mouse	Adult	33,528,710	27,553,478	Han G, Cho C Series GSE175633 (2021)
SRR15597784	Testis	mouse	Adult	45,471,010	37,779,187	

Table S3 in-silico prediction analysis for candidate variants in each family.

Cohort	Gene Name	Genomic Position: Change (GRCh37/ hg19)	Transcript No.	HGVS		Zygosity/ Inheritance	Insilico Predictions		Alternative Allele Frequency		Clinvar
				Nucleotide change	Amino Acid change		CADD Phred	REVEL	KRGDB (1722 individuals)	GMAF (gnomAD)	
SB218-423	DIAPH1	5-140953563 T-TGGA	NM_005219.4	c.1851_1853dupTCC	p.Pro618dup	Het / Autosomal dominant	6.028	NA	Absent	Absent	Likely Benign
	GPR98	5-90106642 C-T	NM_032119.3	c.15565C>T	p.Pro5189Ser	Het / Autosomal recessive	17.21	0.086	Absent	Exome(0.000004012)	No data
	HSD17B4	5-118814538 G-A	NM_001199291.2	c.519G>A	p.Met173Ile	Het / Autosomal recessive	25.6	0.777	Absent	Exome(0.000003987)	No data
	POU4F3	5-145719552 G-GA	NM_002700.2	c.564dupA	p.Ala189fs	Het / Autosomal dominant	NA	NA	Absent	Absent	No data
	PTPN11	12-112926983 C-G	NM_002834.3	c.1599+4C>G		Het / Autosomal dominant	6.33	NA	Absent	Exome(0.000003979)	No data
	SLC26A4	7-107323901 C-T	NM_000441.1	c.920C>T	p.Thr307Met	Het / Autosomal recessive	28.4	0.872	Absent	Exome(0.00007569) Genome(0.00009555)	Uncertain Significance
	TUBB4B	9-140138040 A-AGT	NM_006088.5	c.*36_*37dupTG		Het / Autosomal dominant	5.708	NA	Absent	Exome(0.0002952) Genome(0.0002229)	No data

Cohort	Gene Name	Genomic Position: Change (GRCh37/ hg19)	Transcript No.	HGVS		Zygosity/ Inheritance	Insilico Predictions		Alternative Allele Frequency		Clinvar
				Nucleotide change	Amino Acid change		CADD Phred	REVEL	KRGDB (1722 individuals)	GMAF (gnomAD)	

SB307-610	DNMT1	19-10271082 G-A	NM_001130823.1	c.1021C>T	p.Arg341Cys	Het / Autosomal dominant	23.2	0.068	Absent	Exome(0.000003977) Genome(0.0001273)	No data
	POU4F3	5-145719733 T-C	NM_002700.2	c.743T>C	p.Leu248Pro	Het / Autosomal dominant	32.0	0.950	Absent	Absent	No data

**Cohort**	**Gene Name**	**Genomic Position: Change (GRCh37/ hg19)**	**Transcript No.**	**HGVS**		**Zygosity/ Inheritance**	**Insilico Predictions**		**Alternative Allele Frequency**		**Clinvar**
				**Nucleotide change**	**Amino Acid change**		**CADD Phred**	**REVEL**	**KRGDB (1722 individuals)**	**GMAF (gnomAD)**	
SB347-679	ADCY1	7-45724654 C-T	NM_021116.2	c.2060C>T	p.Ala687Val	Het / Autosomal recessive	23.0	0.107	Absent	Absent	No data
	HARS2	5-140075180 A-AC	NM_012208.3	c.489dupC	p.Ile164fs	Het / Autosomal recessive	23.4	NA	Absent	Exome(0.00008748)	Uncertain Significance
	POU4F3	5-145719942 G-A	NM_002700.2	c.952G>A	p.Val318Met	Het / Autosomal dominant	31.0	0.936	Absent	Absent	Uncertain Significance
	KLHL18	3-47376288 G-C	NM_025010.4	c.877G>C	p.Val293Leu	Het / Autosomal recessive	26.8	0.8	Absent	Exome(0.000008009)	No data

**Cohort**	**Gene Name**	**Genomic Position: Change (GRCh37/ hg19)**	**Transcript No.**	**HGVS**		**Zygosity/ Inheritance**	**Insilico Predictions**		**Alternative Allele Frequency**		**Clinvar**
				**Nucleotide change**	**Amino Acid change**		**CADD Phred**	**REVEL**	**KRGDB (1722 individuals)**	**GMAF (gnomAD)**	
SB438-852	CDH23	10-73157033 C-CCGAGG	NM_022124.5	c.-35_-31dupAGGCG		Het / Autosomal recessive	17.14	NA	Absent	Exome(0.3943) Genome(0.3921)	No data

GPR98	5-90072351 T-C	NM_032119.3	c.12485T>C	p.Ile4162Thr	Het / Autosomal recessive	23.8	0.179	Absent	Exome(0.00004820) Genome(0.00003185)	Uncertain Singificance
POU4F3	5-145719869 C-A	NM_002700.2	c.879C>A	p.Phe293Leu	Het / Autosomal dominant	23.5	0.913	Absent	Absent	No data
SIX2	2-45233332 T-G	NM_016932.4	c.853A>C	p.Asn285His	Het / Autosomal dominant	24.9	0.458	Absent	Absent	No data

Table S4 Detailed information of molecular study

Plasmid construction, cell culture, and transfection

A human *POU4F3* cDNA clone (ORIGENE, CAT# RC211206) served as a template for *in vitro* mutagenesis. These *POU4F3* cDNA-loaded plasmids were subcloned into the pCMV expression vector using oligonucleotides that introduced Mlul and Asisl restriction sites into the 5' and 3' of the cDNA. As described previously,[1] site-directed mutagenesis was performed to create plasmid construct encoding mutant *POU4F3*. Specifically, in the *POU4F3* p.Ala189Serfs*26 plasmid, 376 bp of the cDNA sequence from the in-frame stop codon to the Myc-DDK codon was deleted, and ligation was subsequently performed. Human embryonic kidney 293T cell (HET293T) line was obtained from American Type Culture Collection (ATCC; Manassas, VA, USA). HEK293T cells were transfected with constructs encoding wild-type and mutant proteins fused to C-terminal Myc-DDK tags using Lipofectamine 3000 transfection reagent (Invitrogen). After transfection for 48 hours, cells were fixed in 4% paraformaldehyde for 15 minutes, permeabilized in phosphate-buffered saline (PBS) containing 0.3% Triton X-100 for 10 minutes, and then blocked in PBS containing 10% donkey serum for 1 hour at 37°C in a humid atmosphere.

Immunohistochemistry

The transfected cells were fixed in 4% paraformaldehyde for 15 min, followed by PBS washing, and the process was repeated three times. The HEK293T cells were incubated at 24°C with primary antibodies (anti-Myc [mouse, cell signaling, #2276,1:4000] or anti-DDK [goat, cell signaling, #14793,1:800, 2 h]) (Sigma Aldrich Corp., St. Louis, MO, USA)] for 2 h and Rhodamine phalloidin (Invitrogen, R415, 1:100) for 1 h. They were then washed three times with PBS (4°C), followed by consecutive incubation with secondary antibodies (anti-Myc; 488 goat anti-mouse, Invitrogen, A-11017, 1:400 for 2 h or anti-DDK; goat-anti-mouse Alexa680, Invitrogen, Seoul, Korea; 1:400 for 2 h) and Rhodamine phalloidin (Invitrogen, R415) 1:100 staining for 1h. After washing three times with PBS, the samples were mounted with DAPI (Vector Laboratories Inc., Burlingame, CA) at room temperature for 90 min. The samples were examined with a laser scanning confocal microscope (Zeiss LSM 510, Carl Zeiss, Germany). The number of cells expressing POU4F3 either in the nucleus or the cytoplasm were measured using the Image J software.

Western blot

The cell line was cultured in DMEM (Gibco, Thermo Fisher Scientific, Waltham, MA, USA) at 37°C and humidified air of a 5% CO_2 incubator. The cells were transiently transfected with PCMV6-myc-DDK entry, PCMV6-POU4F3 wild type-myc-DDK, PCMV-POU4F3 A189S frameshift mutant-myc-DDK, PCMV-POU4F3 L248P-myc-DDK, PCMV-POU4F3 F293L-myc-DDK, and PCMV-POU4F3 V318M-myc-DDK expressing plasmids using jetPRIME reagents (Polyplus-transfection SA, Illkirch-Graffenstaden, France), according to the manufacturer's instructions. Whole proteins were separated using 12.5% sodium dodecyl sulfate-polyacrylamide gel electrophoresis (SDS-PAGE) and transferred to 0.45 μm polyvinylidene difluoride (PVDF) membranes (Millipore; Billerica, MA, USA). The membranes were incubated with 5% skim milk to block nonspecific binding at room temperature for 1 h. Membrane blots were incubated against Myc-tag from Cell signaling Technology (Danvers, MA, USA) and β-actin from Santa Cruz biotechnology (Santacruz, CA, USA). The membranes with bound primary antibodies were incubated with anti-mouse secondary antibodies that were conjugated horseradish peroxidase (HRP) (Santacruz) for 1 h at room temperature. The protein band was detected using chemiluminescence (ATTA, Tokyo, Japan). X-ray films (Agfa, Mortsel, Belgium) were used for detection. β-actin antibodies were used as loading controls. The intensity of bands was measured using the Image J software.

Reverse transcription-Polymerized Chain Reaction (RT-PCR)

To verify *POU4F3* expression in LCLs, reverse transcription PCR was conducted following RNA extraction from these cells. The resulting amplicon size corresponded to the anticipated 246 bp, and subsequent Sanger sequencing with ClustalW alignment analysis revealed alignment with *POU4F3*. Furthermore, to validate *POU4F3* expression in HEK293 cells, reverse transcription PCR was performed post-RNA extraction to ascertain the expected size. The observed amplicon size was also 246 bp, and following Sanger sequencing, ClustalW alignment analysis confirmed alignment with *POU4F3*.

Real-time quantitative reverse transcription-Polymerized Chain Reaction (RT-qPCR)

For validation of the RNA sequencing, expression levels of selected genes were evaluated using RT-qPCR. Total RNA was extracted from the lymphoblastoid cell line using RNeasy Mini Kit (Qiagen Inc., Hilden, Germany). cDNA was synthesized using the PrimeScript Reverse Transcriptase-reagent Kit (TaKaRa, RR037A) according to the manufacturer's protocols. RT-qPCR was performed using a Light-Cycler 480 Instrument II, using the Light Cycler 480 probes master kit (Roche; Indianapolis, IN, USA) and SYBR Premix Ex Taq II (TaKaRa, RR420A). The following primers were used for MYO6 genotyping: forward (5′-CCTGACCACTTAGCAGAGTTGG-3′) and reverse (5′-TTTAATGCAGGCTTCAGCTCGATA -3′). The following primers were used for BMP2 genotyping: forward (5′- TGTATCGCAGGCACTCAGGTCA-3′) and reverse (5′-CCATCCGTTTCTGGTACTTCTTC -3′). The following primers were used for AHI1 genotyping: forward (5′-GCTCAGTAGACACAGAACCTGG-3′) and reverse (5′-CTCCTGCATTTAGTGAGAAGAGG-3′). The relative gene expression was calculated using the 2-ddCt analysis method with GAPDH as the endogenous control.

Luciferase assay

HEK293T cells were transfected with the −711 bp upstream SNAP-25 promoter region (**Fig. S2**), including KpnI (GGTAC/C) and XhoI (C/TCGAG) sites, cloned into the pGL4.12[luc2CP] vector (Promega) with pCMV6 vector (Myc-DDK), pCMV6-wt POU4F3 cDNA, pCMV6-p.Ala189Serfs*26 POU4F3 cDNA, pCMV6-p.Leu248Pro POU4F3 cDNA, pCMV6-p.Phe293Leu POU4F3 cDNA, and pCMV6-p.Val318Met POU4F3 cDNA. Forty-eight hours later, the cells were lysed with luciferase cell lysis buffer (200 μL) and luciferase activities were measured under minimal ceiling effect conditions, according to the manufacturer's protocol (Promega, Madison, WI, USA). Experiments were performed in duplicate, and measurements were performed three times to identify Luciferase activity.

Table S5 RNA sequencing and bioinformatic analysis

The RNA sequencing library was prepared and sequenced by Macrogen (Seoul, Korea). Further analyses were done by the Genomics Core Facility in Seoul National University Hospital (Seoul, Korea). RNA sequencing libraries were generated in accordance with the manufacturer's protocols (TruSeq Stranded TotalRNA LT sample prep kit; Illumina). After confirming library size, approximately 400–500 bp, paired-end sequencing was performed. Average total reads were 121,382,372 with over 95% of Q30 reads. For mapping and alignment, raw data were trimmed using the Trimmomatic program to remove adaptor sequences[2]. In addition, a window size of 4, mean quality of 15, and minimum length < 36 bp were set for trimming. Trimmed reads were mapped against the reference genome (GRCh37) using the HISAT2 program[3]. Over 95% of the processed reads were mapped and aligned using the Stringtie program to acquire transcript quantification[4]. In some cases, raw reads were analyzed using the Kallisto program as indicated.[5] Detailed information about the sequencing summary is presented in **Table S2**. Individual samples were further analyzed to achieve the DEG list using the DESeq2 program[6] with RLF normalization and nbinomWaldtest to list fold change over 2, and a p-value < 0.05. For the correlation analyses, normalized reads were used to calculate the Spearman's coefficient. DEGs were further analyzed using Hierarchical and *K*-mean clustering with Euclidean distance and average linkage, and visualized as a heatmap using either Morpheus or the Multiple Experiment Viewer software (MeV, v4.9.0)[7]. The Protein-Protein Interaction (PPI) analysis was performed using the Search Tool Retrieval of Interacting Genes/Proteins (STRING) v11.5 database[8]. Using the DEG list, g: profiler[9] or DAVID[10] ontology analyses were performed to examine the GO terms of biological process, cellular component, molecular function, and the KEGG pathway. Enriched GO terms were further analyzed using ReviGo[11] or QuickGo[12].

References

1 Lee, S. Y. *et al.* Novel genotype–phenotype correlation of functionally characterized LMX1A variants linked to sensorineural hearing loss. *Human Mutation* **41**, 1877-1883 (2020).

2 Bolger, A. M., Lohse, M. & Usadel, B. Trimmomatic: a flexible trimmer for Illumina sequence data. *Bioinformatics* **30**, 2114-2120 (2014).

3 Kim, D., Langmead, B. & Salzberg, S. L. HISAT: a fast spliced aligner with low memory requirements. *Nature methods* **12**, 357-360 (2015).

4 Pertea, M. *et al.* StringTie enables improved reconstruction of a transcriptome from RNA-seq reads. *Nature biotechnology* **33**, 290-295 (2015).

5 Bray, N. L., Pimentel, H., Melsted, P. & Pachter, L. Near-optimal probabilistic RNA-seq quantification. *Nature biotechnology* **34**, 525-527 (2016).

6 Love, M. I., Huber, W. & Anders, S. Moderated estimation of fold change and dispersion for RNA-seq data with DESeq2. *Genome biology* **15**, 1-21 (2014).

7 Saeed, A. *et al.* TM4: a free, open-source system for microarray data management and analysis. *Biotechniques* **34**, 374-378 (2003).

8 Szklarczyk, D. *et al.* STRING v11: protein–protein association networks with increased coverage, supporting functional discovery in genome-wide experimental datasets. *Nucleic acids research* **47**, D607-D613 (2019).

9 Reimand, J., Kull, M., Peterson, H., Hansen, J. & Vilo, J. g: Profiler—a web-based toolset for functional profiling of gene lists from large-scale experiments. *Nucleic acids research* **35**, W193-W200 (2007).

10 Huang, D. W., Sherman, B. T. & Lempicki, R. A. Systematic and integrative analysis of large gene lists using DAVID bioinformatics resources. *Nature protocols* **4**, 44-57 (2009).

11 Supek, F., Bošnjak, M., Škunca, N. & Šmuc, T. REVIGO summarizes and visualizes long lists of gene ontology terms. *PloS one* **6**, e21800 (2011).

12 Binns, D. *et al.* QuickGO: a web-based tool for Gene Ontology searching. *Bioinformatics* **25**, 3045-3046 (2009).
